# Oral sodium hyaluronate improves skin hydration, barrier function and signs of aging: a randomized, double-blind, placebo-controlled trial in 150 healthy adults

**DOI:** 10.1038/s41598-025-32758-5

**Published:** 2025-12-20

**Authors:** Iva Dolečková, Pavel Kušnierik, Vratislav Berka, Matěj Šimek, Ilona Matějková, Filip Prokopec, Petra Ovesná

**Affiliations:** 1 Contipro a.s., Dolní Dobrouč 401, Dolní Dobrouč, 561 02 Czech Republic; 2 Institute of Biostatistics and Analyses, Faculty of Medicine, Masaryk University, Brno, Czech Republic

**Keywords:** Nutraceutical, Oral sodium hyaluronate, Randomized controlled trial, Skin hydration, Transepidermal water loss, Wrinkles, Diseases, Health care, Medical research

## Abstract

Oral hyaluronan has been reported to improve various aspects of skin physiology, but existing trials often lack methodological rigor, comprehensive outcome assessment, and diverse populations. This randomized, double-blind, placebo-controlled trial evaluated the effects of sodium hyaluronate (SH) supplementation at two daily doses on skin parameters in healthy Caucasian adults. A total of 150 participants were randomized to receive SH (1.8 MDa) at 60 mg/day (SH60), 120 mg/day (SH120), or placebo for 12 weeks. Facial hydration, transepidermal water loss (TEWL), sebum, elasticity, wrinkle depth, skin gloss, colorimetric parameters, epidermal thickness, dermal density, red areas, and pore size were assessed at baseline and monthly. Subjective skin condition was evaluated every two weeks, and components of natural moisturizing factor (NMF) in forearm skin were quantified by LC-MS/MS. SH120 significantly enhanced skin hydration and elasticity, while reducing TEWL, sebum, and periorbital wrinkle depth versus placebo. It also improved skin structure by increasing epidermal thickness, dermal density, and NMF levels. SH60 showed similar but more modest effects. No changes were observed for colorimetric parameters, red areas, pore size, or gloss. In conclusion, oral SH supplementation improved multiple aspects of skin physiology, supporting its use as a functional food ingredient with measurable benefits for skin health and healthy aging.

Trial registration: ClinicalTrials.gov, NCT07065110 (retrospectively registered on 15 Jul 2025); EFSA (European Food Safety Authority) registry: EFSA202400027979 (prospectively filed on 06 Jun 2024).

**Supplementary Information:**

The online version contains supplementary material available at 10.1038/s41598-025-32758-5.

## Introduction

Hyaluronan is a glycosaminoglycan found throughout the body that plays a vital role in various biological processes, including cell signaling, wound healing, tissue regeneration, morphogenesis, and extracellular matrix organization^1^. Due to its excellent biocompatibility, biodegradability and non-immunogenicity, hyaluronan, usually in the form of sodium hyaluronate (SH), has been used in a wide range of medical, pharmaceutical, cosmetic and dietary applications^1^.

In recent years, orally administered SH as a dietary supplement has gained significant attention for its potential health benefits, particularly in relieving chronic knee pain^2^ and improving skin condition, especially hydration as summarized by Sun et al.^3^.

However, despite the growing body of evidence, the overall quality of these studies remains variable. Among the limitations are small sample sizes, specific skin conditions (e.g. dry, aged skin), absence of detailed statistical analyses, or publication solely in Japanese, which reduces accessibility for international evaluation. Also, while skin hydration has been the primary focus of most studies followed by wrinkles and elasticity, other critical parameters such as transepidermal water loss (TEWL), sebum levels, and skin color metrics have been insufficiently explored despite their relevance for evaluating overall skin health and appearance. Moreover, most previous studies were conducted in Asian cohorts, limiting generalizability to other populations. Only one trial evaluated Caucasian participants exclusively^4^. Most prior studies also used relatively high daily doses of SH (100–240 mg), despite emerging evidence suggesting efficacy at substantially lower dosages (e.g., 25–80 mg/day) in other therapeutic areas^2,5^.

These shortcomings in study design, population representativeness, and dose justification highlight the need for well-designed randomized controlled trials (RCTs) that adhere to internationally recognized standards such as the SPIRIT guidelines^6^. There is also a clear need to evaluate a broader range of objective skin parameters beyond hydration, in order to comprehensively understand the dermatological effects of oral SH supplementation.

In response to this need, we conducted a 12-week, double-blind, placebo-controlled RCT to evaluate the effects of oral supplementation with SH (1.8 MDa) at two daily dosages: 120 mg (SH120), a standard dose used in previous studies, and 60 mg, a lower dose not previously tested. The study was conducted in a healthy adult Caucasian population with Fitzpatrick skin phototypes I–III. The primary endpoint was skin hydration after three months of SH supplementation on the cheek, secondary endpoints included a comprehensive set of skin-related outcomes: TEWL, sebum content, elasticity, wrinkle depth, skin gloss, various colorimetric parameters, epidermal thickness, dermal density (collagen content), redness areas, pore size, and hydration at remaining time points and sites. In addition, we measured levels of selected hygroscopic compounds in the stratum corneum (SC; e.g., urea, amino acids and their derivatives) and self-reported data on multiple skin aspects (e.g., hydration, oiliness, smoothness, skin sensitivity), rating both the current state and perceived change relative to baseline. This comprehensive approach aimed to provide robust evidence on the systemic dermatological benefits of SH supplementation in a Western population.

## Materials and methods

### Study design, objectives and registration

This was a single-center, double-blind, randomized, placebo-controlled, parallel-group clinical trial conducted in accordance with the SPIRIT 2013 guidelines^6^. The primary objective was to evaluate the superiority of orally administered SH compared to placebo in improving facial skin hydration on the cheek in healthy adult Caucasian participants after 3 months of supplementation. The study followed a superiority framework.

Secondary objectives included evaluating the effects of SH on the skin hydration at remaining time points and sites, on other skin parameters (TEWL, sebum level, elasticity parameters, wrinkle depth, skin gloss, colorimetric parameters (erythema and melanin indices, ITA°, a* and b* values), pore size and redness areas, epidermal thickness, and dermal density), and on subjective perception of the skin condition (e.g., hydration, sensitivity, oiliness) and its changes from baseline, as well as on the composition of the natural moisturizing factor (NMF) in SC.

The study was registered in the EFSA (The European Food Safety Authority) trial registry (ID: EFSA-2024–00027979) prior to participant enrollment. The trial was then registered retrospectively at ClinicalTrials.gov (ID: NCT07065110) in accordance with journal transparency requirements.

The study was conducted at the facilities of Contipro a.s. (Dolní Dobrouč, Czech Republic). Eligible participants were recruited between June and August 2024. The intervention and clinical assessments were performed from September to December 2024. Data processing, statistical analysis, and interpretation took place from January to June 2025.

### Ethical considerations

The study was conducted in accordance with the Declaration of Helsinki (2013 revision) and the study protocol was reviewed and approved by the Contipro Research Ethics Committee prior to study initiation (ID: AICE24_II, approved on 13th June 2024). Written informed consent was obtained from all participants before initiation of the study. Participants were allowed to withdraw from the study at any time without providing a reason. Given the minimal risk profile of the intervention, no adverse events were anticipated. Participants were monitored throughout the study and had the option to consult medical staff if needed. Data confidentiality was ensured through anonymization and secure data handling in compliance with the General Data Protection Regulation (EU 2016/679).

### Sample size calculations

The study was scheduled from autumn to winter, a period associated with reduced air humidity and an expected seasonal decline in cheek skin hydration of approximately 8.6% in the placebo group^7^. Several clinical trials have shown that oral SH supplementation can improve skin hydration, with group differences at study endpoints between approximately 7–30%^4,8–16^.

The sample size calculation was based on an expected between-group difference of 5 AU (≈ 8.6%) in cheek hydration after 3 months, assuming a conservative standard deviation of 15%. To detect this effect with 80% power and a two-sided alpha of 0.05 (two-sample t-test), 49 participants per group were required. Calculations were performed using R software (version 4.3.2). With an estimated 2% dropout rate, 50 participants per group (150 total) were enrolled.

### Participant eligibility criteria, recruitment and randomization

Healthy adult volunteers were screened for eligibility via an online questionnaire. Inclusion criteria were: age 18–60 years, Caucasian ethnicity, Fitzpatrick skin phototype I–III, and absence of acute or chronic skin or gastrointestinal disorders, willing to maintain their usual skincare routine, avoid other dietary supplements containing SH, avoid excessive UV exposure, avoid longer stays in significantly different climates (assessed on a case-by-case basis) for the duration of the study.

Exclusion criteria included pregnancy or lactation, known allergies to any component of the tested products, use of antibiotics or SH-containing supplements within three months before study initiation, and diabetes mellitus. The first participants enrolled were prioritized to ensure age and sex balance, individuals from saturated strata were excluded once quotas were met.

Participants were stratified by sex (male/female) and age group (18–39 and 40–60 years), and randomly assigned to receive either the SH dietary supplements (lower concentration, daily dose 60 mg (SH60); and higher concentration, daily dose 120 mg (SH120)) or placebo in a 1:1:1 ratio. Randomization was performed using the RAND() function in Microsoft Excel within each stratum to ensure balanced distribution.

### Interventions and placebo justification

Participants obtained altogether 3 flasks containing 500 mL of SH60, SH120 or placebo solution. They took 15 mL of the assigned solution once daily in the morning for 3 months. Characterization of SH60, SH120 and placebo solutions is shown in Table S1. The 3 solutions were indistinguishable in appearance, taste and smell and packaged in identical opaque flasks. SH (Nutrihyl^®^, food grade) used in this study was of microbial origin (*Streptococcus equi* subs. *zooepidemicus*), manufactured by Contipro a.s. (Czech Rep.) and had molecular weight (Mw) of 1.8 MDa. Details about the characterization and microbial safety of the tested solutions can be found in the Supplementary material.

Xanthan gum was chosen as placebo. While there are studies indicating potential effects of xanthan gum on the gut microbiome or overall health, many of these studies are either conducted in vitro^17^ or involve significantly higher doses (e.g., 10 g^18^ or 12 g daily^19^ than the dosage we intended to administer (max 0.1 g daily). Moreover, xanthan gum is a widely used food additive found in various consumer products in Europe^20^ suggesting that the gut microbiome of most individuals is probably adapted to its presence. As such, the introduction of xanthan gum as a placebo was less likely to significantly affect the gut microbiome compared to less common additives.

Participant adherence was assessed by self-reported missed doses, recorded biweekly in the questionnaire, and by weighing returned bottles containing residual solutions at each visit. Average daily intake (mL) was calculated from the weight difference of supplement bottles at each study visit (assuming 1.0 g/mL density).

### Blinding and allocation concealment

The study was conducted in a double-blind manner. Both participants and outcome assessors were blinded to the intervention allocation throughout the study. The random allocation sequence was generated by a designated investigator not involved in participant enrollment or data collection, using the RAND() function in Microsoft Excel to predefine the random ordering of intervention codes. The code key linking intervention codes to group assignments was kept exclusively by this investigator in a secure electronic file inaccessible to study personnel enrolling participants or collecting data until study completion. This investigator received stratified, anonymized participant codes from laboratory staff responsible for participant enrollment and data collection, and performed randomization within each stratification block using the RAND() function.

Laboratory staff responsible for enrollment, data collection, and skin sampling created the stratified, anonymized list of participants, and provided it to the designated investigator responsible for randomization. They then received this list back with preassigned supplement identifiers to distribute the flasks accordingly. Personal identifying data of participants were stored separately in an electronic file that was inaccessible to the investigator responsible for randomization until study completion.

Unblinding was permissible in the event of a serious adverse event. In such cases, the use of unique identifier codes ensured that blinding was maintained for other participants and study personnel.

### Outcome measures

#### Measurement of the skin parameters

Skin parameters were measured on the right cheek and the center of the forehead; skin hydration was additionally assessed on the volar forearm. Epidermal thickness and dermal density were evaluated on the forehead only. Wrinkle depth was evaluated in the crow´s feet area. All measurements were performed under standardized environmental conditions (temperature: 20–22 °C; relative humidity: 40–50%) following at least 30 min of acclimatization. Measurements were conducted at baseline (T0) and after 1, 2, and 3 months of intervention.

SC hydration was assessed using a Corneometer CM 825 (artificial units, AU, 10 values)^21^, TEWL by a Tewameter TM 300 (g/m^2^/h, at least 10 values after saturation of the tewameter chamber)^22^, sebum content by a Sebumeter SM 815 (static measurement, µg/cm^2^, 1 value)^23^, erythema and melanin indices by a Mexameter MX 18 (AU, at least 5 values)^24^, skin color parameters (ITA° (°), a*, b* values (unitless)) by a Colorimeter CL 400 (at least 5 values)^24^, skin gloss by a Glossymeter GL 200 (gloss units, GU, at least 5 values) (all Courage&Khazaka, Germany). Skin elasticity was determined by a Cutometer MPA 580 (aperture 2 mm, 5 repetitions per measurement, 3 measurements) providing parameters R0–R9 (R0, R1, R3: mm, R2, R4-R9: unitless) calculated from deformation-time curves using standard algorithms^25^. A detailed description of each parameter is shown in Figure S1. Parameter R8 was not included in the analysis as it denotes the total recovery amplitude which is not normalized to initial deformation. Therefore, it is not commonly used as a stand-alone elasticity index. Wrinkle depth (µm, 3 values) was determined using Primos Lite (Canfield Scientific, USA) based on 3D fringe projection^26^. High-resolution 3D images of the face were captured using Visia-CR (Canfield Scientific, USA) to evaluate redness areas (pix^2^), and pore size (pix^2^) using ImagePro image analysis software (Media Cybernetics, USA)^27^. Epidermal thickness (µm) and dermal density (% of echogenic pixels) was assessed by high-frequency (22 MHz) ultrasound using the Ultrascan UC22 (Courage&Khazaka, Germany)^28^.

#### Questionnaire

Subjective evaluation of facial skin condition was performed using a structured self-assessment questionnaire administered every 2 weeks (Figure S2). The questionnaire also included safety monitoring items, such as the missed doses, the occurrence of adverse effects or other unintended effects, and any external events potentially affecting the skin or gastrointestinal condition (e.g., illness, sun exposure, travel).

Participants rated their perception of skin hydration, roughness, oiliness, elasticity, wrinkle number and depth, and skin sensitivity using five-point Likert-type scales. Each skin attribute was assessed on a scale ranging from 1 (e.g., “Hydrated”; “No wrinkles”) to 5 (e.g., “Dry”, “Many wrinkles”). Data were processed by calculating the mean score for each parameter at each time point. The values were then expressed as differences from baseline (T0).

Additionally, participants reported perceived changes in these parameters compared to baseline using a five-point qualitative scale (e.g., “Significantly more hydrated” to “Significantly drier”). The percentage of participants selecting each response category was calculated.

Subjective questionnaire data were analyzed descriptively to identify trends and patterns in perceived skin condition. No statistical testing was performed, as these results were intended only to support and contextualize the objectively measured outcomes.

#### Skin sample collection and NMF analysis

SC samples were obtained from the volar forearm using a standardized tape-stripping procedure, as previously described^29^. Five D-Squame sampling discs (1.4 cm diameter; CuDerm, USA) were sequentially applied to the same skin site under uniform pressure. The first three strips were discarded, and the final two discs were pooled and stored at -80 °C until further analysis.

Extraction was performed using 30% methanol with 1% trifluoroacetic acid, spiked with a diluted internal standard (cell free amino acid mixture-13C, 15N, cat. no. 767964-1EA, Sigma-Aldrich, USA). After vortexing, samples were mixed with acetonitrile (4:1, v/v), centrifuged, and filtered through nylon syringe filters prior to analysis. NMF components were quantified using liquid chromatography-tandem mass spectrometry (LC-MS/MS). Chromatographic separation was achieved on an ACQUITY Premier BEH Amide column (1.7 μm, 2.1 × 50 mm), with a mobile phase consisting of 10 mM ammonium formate and 0.125% formic acid in water (a) and in 95% acetonitrile (b)^30^. The selected reaction monitoring (SRM) mode used for detection was developed in-house by direct injection of internal standards. The injection volume was 3 µL. Twenty-three NMF-related molecules were detected and quantified using 10-point external calibration (range: 50–7500 ng/mL). Quantification was based on area ratios of analyte to internal standard. Samples were injected in random order with regular quality and blank controls. Each sample was analyzed in duplicate. Data were processed using TraceFinder software (version 5.1, Thermo Fisher Scientific; https://www.thermofisher.com/cz/en/home/industrial/mass-spectrometry/liquid-chromatography-mass-spectrometry-lc-ms/lc-ms-software/lc-ms-data-acquisition-software.html#tracefinder), and peak areas were exported to Microsoft Excel.

In addition to the analysis of individual NMF components, two NMF-related summary values were calculated: (1) total NMF, defined as the sum of all quantified components, and (2) filaggrin-derived NMF, including only amino acids and metabolites primarily resulting from filaggrin degradation (i.e., all compounds excluding urea, citrulline, and ornithine). For each participant, the values were normalized to individual baseline levels and expressed as percentage of baseline (%T0).

### Data analysis

Statistical analyses were performed using Microsoft Excel, QC Expert (version 3.3.0.6, TriloByte Ltd., Czech Republic) and R software (version 4.3.2). The full analysis set (FAS) included all randomized participants with available primary outcome data. No imputation was performed, as missing data did not exceed 10%. Unless stated otherwise, data were normalized to baseline values and expressed as percentage of baseline (%T0). Normality of data distribution was assessed using the Kolmogorov-Smirnov test and supported by visual inspection of histograms and Q-Q plots where appropriate. Depending on the distribution, either parametric (independent sample t-test) or non-parametric (Mann–Whitney U test) statistical methods were applied. Results are presented as mean ± confidence intervals (CI), unless indicated otherwise. Comparisons of the SH60 and SH120 groups to placebo were performed at a significance level of α = 0.05. *P* values < 0.05 were considered statistically significant. No correction for multiple comparisons was applied, as each outcome represented a distinct and independently measured skin parameter with a different biological target.

### Declaration of generative AI and AI-assisted technologies in the writing process

The authors acknowledge the use of ChatGPT to assist in refining the English language. All content was critically reviewed to ensure scientific accuracy and integrity.

## Results

### Participant characteristics, adherence, adverse events

**Fig. 1:**
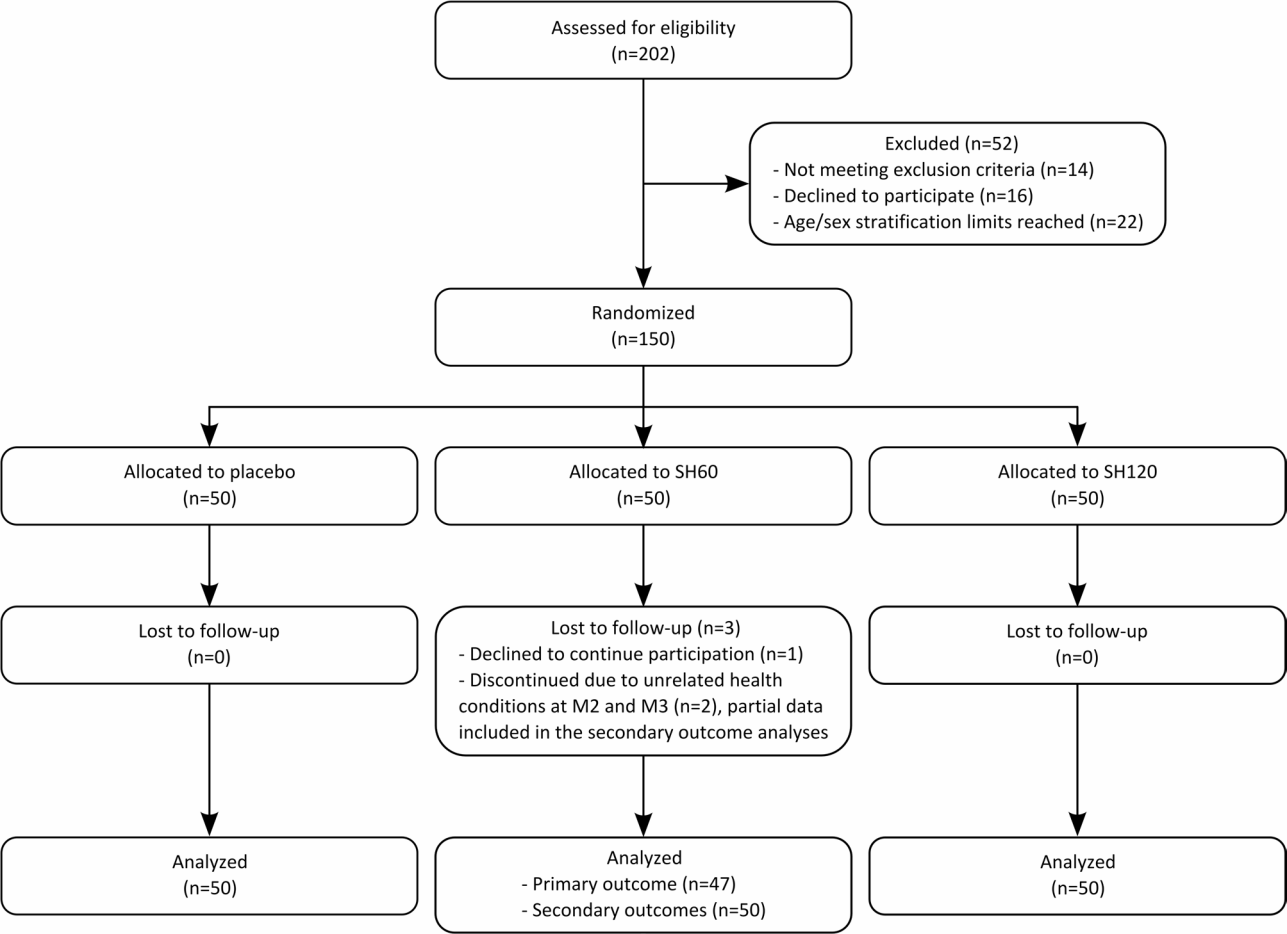
Participant flow diagram.

A total of 202 individuals were screened for eligibility, of whom 150 were randomized into three study groups (placebo, SH60, and SH120; 50 individuals per group, 1:1:1 allocation ratio) (Fig. 1). All randomized participants initiated the intervention. No participants were lost to follow-up in the placebo or SH120 groups. In the SH60 group, three participants discontinued the study prematurely: one withdrew consent, and two discontinued due to unrelated health conditions after two and three months (one participant with pre-existing vitiligo decided to initiate systemic biologic therapy during the study period, and another developed a respiratory infection requiring antibiotic treatment). These two participants contributed partial data and were included in secondary outcome analyses, but not in the primary outcome analysis. Baseline characteristics of participants included in the primary outcome analysis are summarized in Table 1. The groups were balanced in terms of sex distribution and age.

**Table 1:** Baseline characteristics of participants included in the primary outcome analysis.

	Placebo	SH60	SH120
Number	50	47	50
Female/male	32/18	31/16	32/18
Age, mean ± SD	43.4 ± 10.8	44.3 ± 8.8	43.8 ± 9.2
Age, range	18–60	22–59	24–60
Fitzpatrick phototype I/II/III	3/45/2	2/43/2	3/44/3

Adherence to the supplementation regimen was high in all groups. The mean number of missed doses per participant was low, ranging from 1.5 to 2.2 doses over the course of the study and the reported daily intake of the test product remained close to the target of 15 mL throughout the intervention (Table S2). These results suggest good compliance with the study protocol across all groups.

A variety of mild adverse events such as worsening of skin and hair condition (Table S3) as well as spontaneously self-reported positive effects (Table S4), including improvements in hair condition, skin quality, or joint comfort, were reported across all groups, including placebo. The frequency and distribution of these events were comparable across groups and did not indicate any treatment-related pattern.

### Skin parameters

Due to the large number of measured parameters evaluated at different anatomical sites, only selected outcomes showing statistically significant differences or clinically relevant trends are visualized in the main text. Complete aggregated datasets of all measured parameters, including raw values and changes relative to baseline (%T0), are provided in Table S5. Corresponding *p* values for between-group comparisons (SH60 and SH120 vs. placebo) are presented in Table S6.

**Fig. 2:**
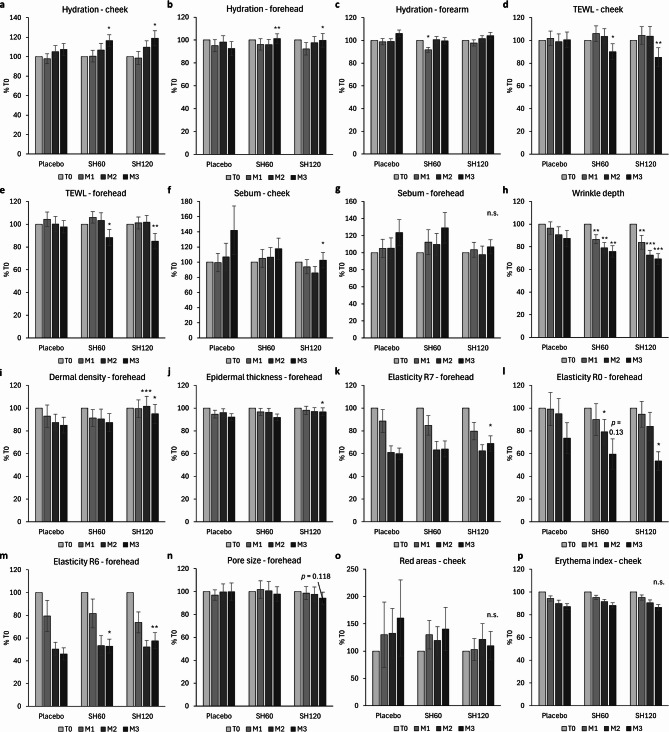
Effect of oral supplementation with SH60 and SH120 on selected skin parameters. (**a**) Hydration on the cheek; (**b**) hydration on the forehead; (**c**) hydration on the volar forearm; (**d**) transepidermal water loss (TEWL) on the cheek; (**e**) TEWL on the forehead; (**f**) sebum on the cheek; (**g**) sebum on the forehead; (**h**) periorbital wrinkle depth; (**i**) dermal density (collagen content) on the forehead; (**j**) epidermal thickness on the forehead; (**k**) R7 (biological elasticity) on the forehead; (**l**) R0 (skin pliability) on the forehead; (**m**) R6 (viscous component of skin mechanical behavior) on the forehead; (**n**) pore size on the forehead; (**o**) red areas on the cheek; (**p**) erythema index on the cheek. Data are presented as percentage of baseline (%T0; mean ± 95% CI). *p < 0.05, **p < 0.01 vs. placebo.

The primary outcome was the change in skin hydration on the cheek after three months of SH supplementation. A statistically significant increase was observed in both the SH60 and SH120 groups (+ 9.1% and + 11.5%, respectively) compared to placebo (Fig. 2a). A slight time- and dose-dependent increasing trend was noted. In addition, a significant improvement in hydration in comparison to placebo was found on the forehead after three months in both intervention groups (+ 8.7% for SH60 and 7.2% for SH120) (Fig. 2b). No consistent time- or intervention-dependent changes were observed on the volar forearm (Fig. 2c).

A significant reduction in TEWL was shown at both the cheek and forehead after three months of supplementation in the SH60 and SH120 groups in comparison to placebo suggesting enhanced skin barrier (Fig. 2d and e).

A marked increase in sebum levels was found in the placebo group at both the cheek and forehead sites after three months (Fig. 2f and g). This change was significantly attenuated in the SH120 group at both locations, and a slight, non-significant decrease was noted in the SH60 group on the cheek as well.

**Fig. 3:**
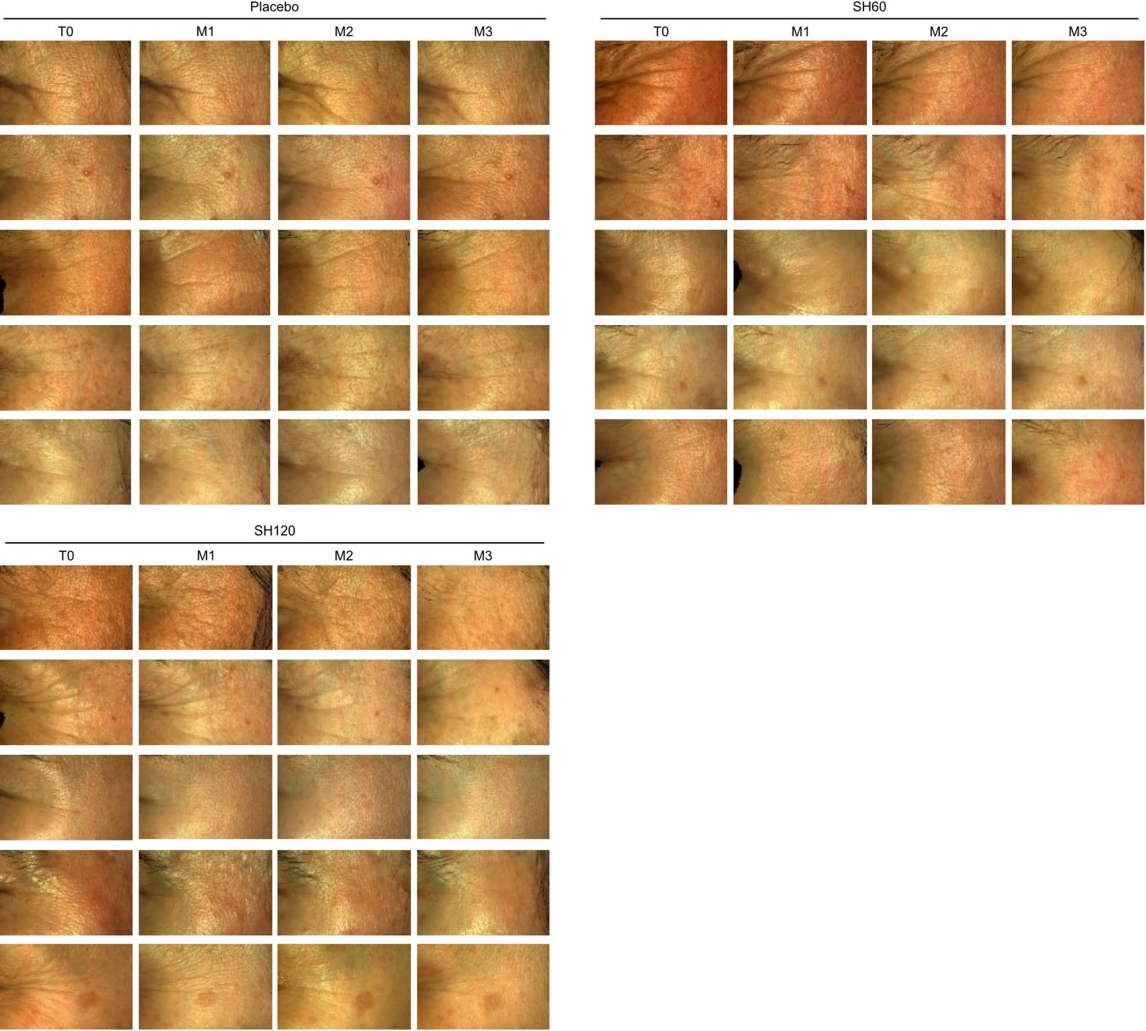
Illustrative examples showing the effect of oral supplementation with SH60 and SH120 on periorbital wrinkles over time. Noticeable reduction in the SH60 and SH120 groups can be observed whereas only minimal changes in the placebo group are visible.

A statistically significant reduction in periorbital wrinkle depth was observed in both the SH60 and SH120 groups compared to placebo as early as one month after the start of supplementation (Fig. 2h). This reduction was readily visible in high-resolution images (Fig. 3). A slight wrinkle reduction was also noted in the placebo group.

**Fig. 4:**
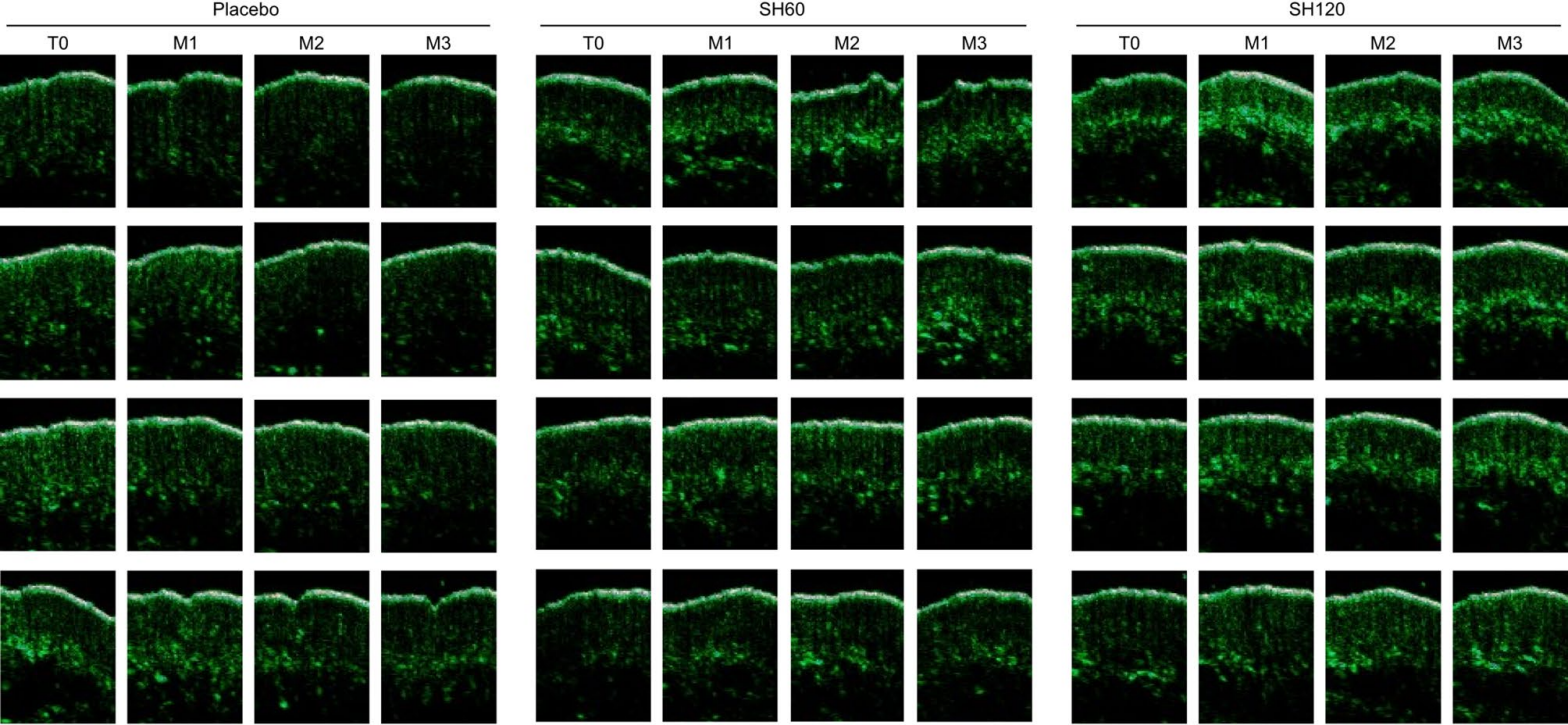
Illustrative examples showing the effect of oral supplementation with SH60 and SH120 on dermal density (collagen level, green signal) visualized by ultrasound imaging. A progressive decrease in dermal density is visible in the placebo group, whereas no decrease, or even a slight increase, is observed in the SH60 and SH120 groups.

Ultrasonographic analysis of skin structure on the forehead revealed a gradual decrease in dermal density corresponding to collagen level in the placebo group, which was significantly attenuated in the SH120 group after two and three months as shown in graph (Fig. 2i) as well as in representative images (Fig. 4).

Epidermal thickness followed a similar pattern, with a subtler, progressive decrease observed in the placebo group that was significantly less pronounced in the SH120 group (Fig. 2j).

Among the parameters obtained from the viscoelastic curves from the cutometer device (see Figure S1 for a detailed description), R2 (gross elasticity), R5 (net elasticity), and R7 (biological elasticity) reflect the ability of the skin to return to its original shape after deformation with higher values indicating better elasticity. All three parameters significantly declined throughout the study at both the cheek and forehead, suggesting seasonal deterioration in skin elasticity. A representative result for R7 on the forehead is shown in Fig. 2k; remaining results for these parameters at both anatomical sites are presented in Figure S3. In the case of R5 and R7, the decline was slightly but significantly attenuated in the SH120 group compared to placebo on the forehead, indicating increased skin elasticity. No consistent intervention-related differences were observed for R2.

Parameters R0, R1, R3, R4, and R9 are associated with skin distensibility and mechanical fatigue, where lower values reflect enhanced skin firmness and resilience. Results for R0 on the forehead are shown in Fig. 2l, remaining results can be found in Figure S3. All these parameters declined over the course of the study, suggesting greater skin resilience and an improved ability to recover after deformation. A more pronounced, significant decrease in R0 and R3, and a similar, non-significant decreasing trend in R1 and R4 were observed in the SH120 group compared to placebo on the forehead after three months. A similar decreasing trend was also shown in the SH60 group on the forehead. These findings suggest enhanced skin resilience and reduced “tiring” effect associated with more elastic skin following SH supplementation. No between-group differences were observed in R9 parameter (Figure S3).

R6 reflects the viscous component of skin mechanical behavior. Results for R6 are shown in Fig. 2m (forehead), and in Figure S3 (cheek). During the study, R6 progressively decreased in all groups, indicating a general shift toward less viscous skin. This decrease was attenuated in both SH60 and SH120 groups, with slightly, but significantly higher R6 values compared to placebo at three months. A similar trend was observed in the SH120 group on the cheek, although the results were on the significance threshold (*p* = 0.052). This suggests that SH supplementation partially increased the viscous component of skin mechanical properties, possibly reflecting softer or more hydrated skin.

A slight decreasing trend in pore size close to the significance threshold was observed during the study on the forehead in the SH120 group compared to placebo (Fig. 2n), whereas no consistent time- or dose-dependent trends were detected on the cheek (Figure S3).

**Fig. 5:**
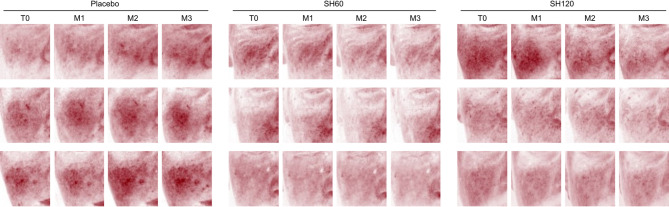
Illustrative examples showing the effect of oral supplementation with SH60 and SH120 on cheek redness over time visualized under cross-polarized light. Increased facial redness is visible in the placebo group, whereas no increase, even a slight reduction, is observed in the SH60 and SH120 groups.

An increasing trend in facial redness areas was noted in the placebo group over the study period on both the cheek (Fig. 2o) and the forehead (Figure S3), as illustrated by representative images of the cheek in Fig. 5. This increase was visibly attenuated in both SH60 and SH120 groups, although the differences from placebo were not statistically significant. Other redness-related parameters, including the erythema index obtained from the mexameter (cheek: Fig. 2p, forehead: Figure S3) and the a* value measured by the colorimeter (Figure S3) showed a gradual decrease throughout the study, with no notable between-group differences. This reduction, along with a concurrent decline in the melanin index, an increase in ITA° values, and a slight decrease in b* values (yellow hues) across all study groups (Figure S3), likely reflects seasonal skin lightening, typically occurring from late summer/early autumn to winter. SH supplementation had no apparent effect on these colorimetric parameters.

No consistent time-dependent or treatment-related differences in skin gloss were observed across the study groups (Figure S3).

### Subjective assessment of the skin parameters (questionnaires)

**Fig. 6:**
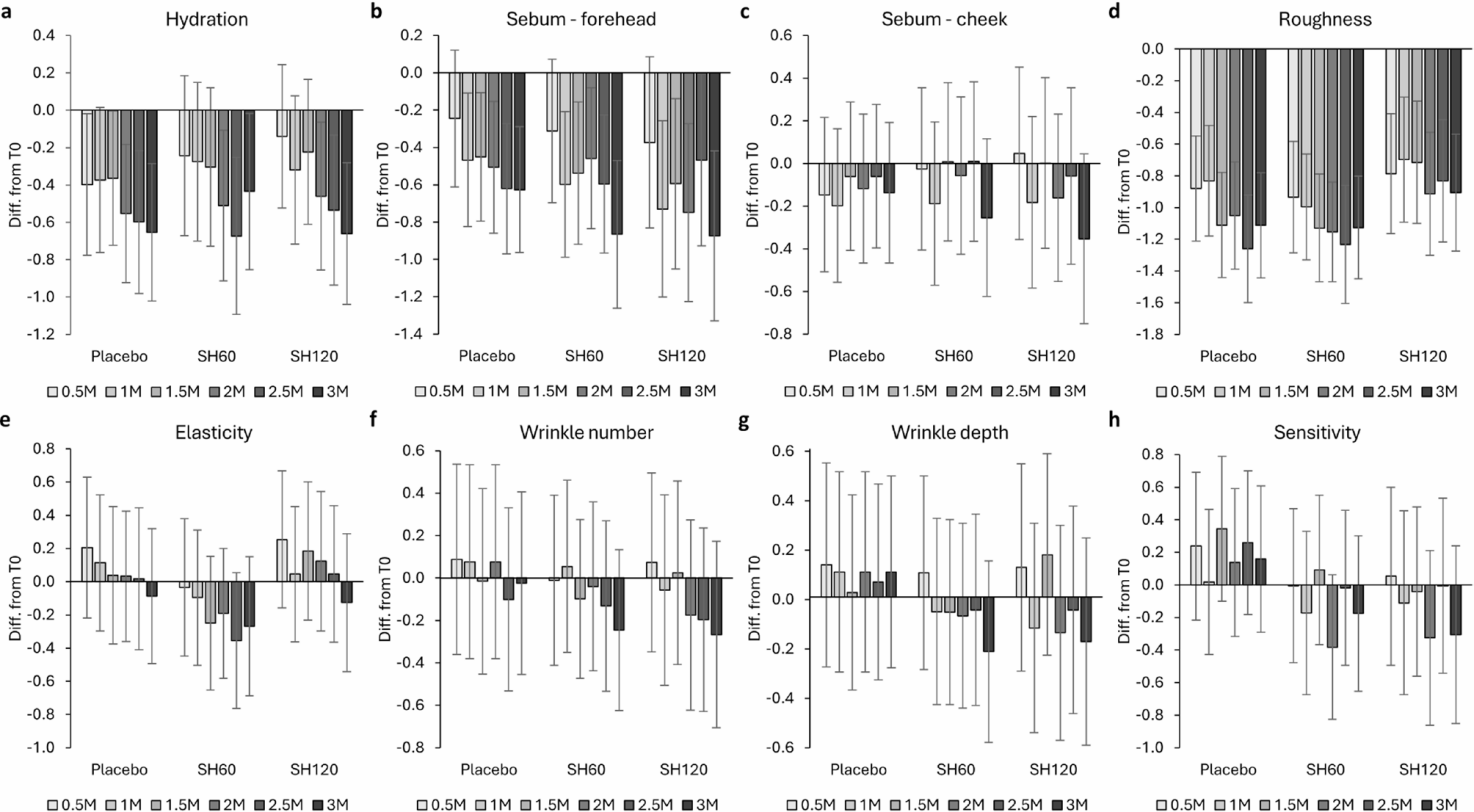
Effect of oral supplementation with SH60 and SH120 on self-reported skin parameters rated on a 5-point scale (questionnaire assessment). (**a**) Skin hydration (5 = dry, 1 = hydrated); (**b**) sebum on cheek (5 = oily, 1 = dry); (**c**) sebum on forehead (5 = oily, 1 = dry); (**d**) roughness (5 = rough, 1 = smooth); (**e**) elasticity (5 = low, 1 = good); (**f**) number of wrinkles (5 = many, 1 = none); (**g**) depth of wrinkles (5 = very deep, 1 = none); (**h**) skin sensitivity (5 = high sensitivity: frequent irritation, burning, or itching; 1 = low sensitivity: never irritated). Values represent mean change from baseline (T0) at each time point (mean ± 95% CI).

In the first part of the questionnaire, participants rated their perception of skin parameters using five-point scales. The calculated mean scores for each parameter at each time point are shown in Table S7, while Fig. 6 displays differences from baseline. Although considerable inter-individual variability was present across groups and time points (as reflected by wide CIs), several trends could be observed.

Participants consistently reported increased skin hydration throughout the study in all groups, including placebo, and no differences were noted between the intervention groups (Fig. 6a). The observed improvement across all groups may reflect a placebo or expectation-related effect and a subjective perception of hydration influenced by factors other than actual water content such as oiliness, skin structure, roughness, or skin sensitivity. Self-assessed oiliness, particularly on the forehead (Fig. 6b), showed a decrease in all groups. This effect was more pronounced in the SH60 and SH120 groups by the end of the study. Cheek oiliness (Fig. 6c) exhibited a subtler downward trend, but a similar, more substantial decrease in the SH60 and SH120 groups compared to placebo was observed after three months. Notably, the perceived reduction in oiliness in SH60 and SH120 groups compared to placebo after three months was consistent with the objectively measured sebum level via the sebumeter. A general reduction in skin roughness was reported within all groups showing improvement (Fig. 6d). However, the SH120 group exhibited a slightly attenuated response compared to placebo and SH60. No coherent trends were detected for skin elasticity, and no treatment-specific effects could be distinguished (Fig. 6e). Both wrinkle number and depth were perceived as slightly improved in the SH60 and SH120 groups, whereas the placebo group showed either no change or a slight worsening (Fig. 6f and g). These results are in agreement with wrinkle reduction determined by 3D skin profiling in both SH60 and SH120 groups compared to placebo. Self-perceived skin sensitivity showed a slight decreasing trend over time in both SH groups, while the placebo group showed a marginal increase (Fig. 6h). These results may be related to the objectively measured improvement of the skin barrier function (TEWL reduction) and slightly reduced facial redness in SH groups possibly reflecting reduction in subclinical irritation.

The second set of questionnaire items assessed participants’ perceived changes in skin parameters compared to baseline. Figure S4 shows the proportion of participants reporting selected changes for each parameter throughout the study. A detailed description of these results is provided in the Supplementary material. This categorical self-assessment of perceived changes from baseline (improvement/worsening) proved more susceptible to placebo-related bias compared with the 5-point scale method, and no consistent differences were detected between groups.

### NMF analysis

Twenty-three NMF-related compounds were quantified, including free amino acids (e.g., serine, glycine, histidine), their derivatives (e.g., pyrrolidone carboxylic acid (PCA)), urea, and urea-related metabolites (citrulline, ornithine). A full list of analytes with their relative proportion is provided in Figure S5. The most abundant NMF constituents were serine, urea, glycine, histidine, citrulline, and threonine. In addition to total NMF defined as the sum of all quantified components, we also analyzed filaggrin-derived NMF, which included all compounds except urea, citrulline, and ornithine.

**Fig. 7:**
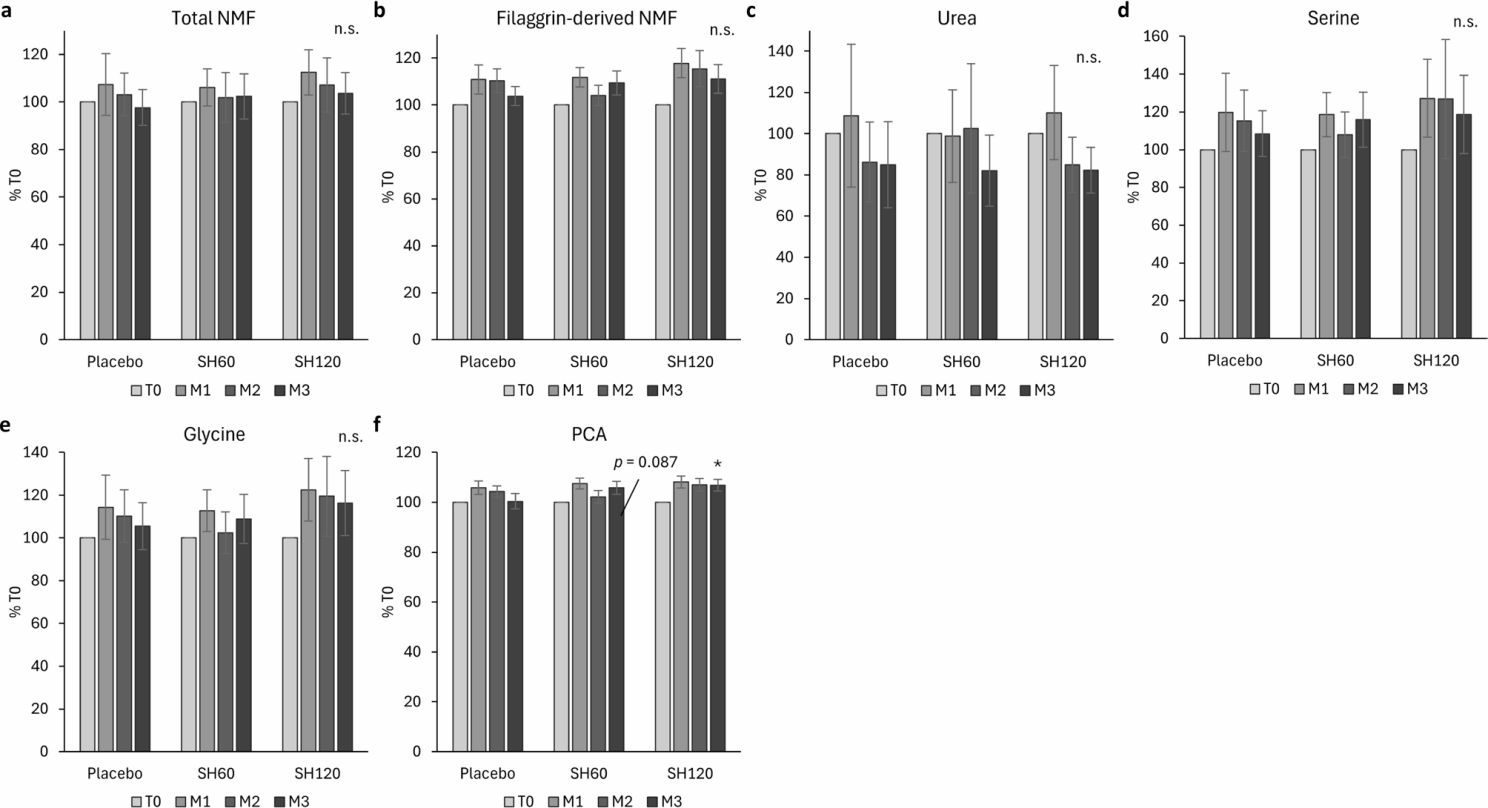
Effect of SH supplementation on natural moisturizing factor (NMF) levels in the stratum corneum of volar forearm skin. (**a**) Total NMF (sum of all quantified components); (**b**) filaggrin-derived NMF (sum of all quantified components excluding urea, citrulline and ornithine); (**c**) urea; (**d**) serine; (**e**) glycine; (**f**) pyrrolidone carboxylic acid (PCA). Data are presented as percentage of baseline (%T0; mean ± 95% CI). *p < 0.05 vs. placebo.

Although no statistically significant differences were found in the concentrations of total NMF or filaggrin-derived NMF components between the SH groups and placebo (*p* > 0.05), slightly higher levels were observed in the SH120 group compared to placebo (Fig. 7a and b).

Analysis of individual NMF components revealed a decrease in urea levels, possibly reflecting seasonal decline in sweat excretion with no treatment-specific differences (Fig. 7c). Levels of serine and glycine, the two most abundant hygroscopic amino acids, followed a filaggrin-derived NMF pattern and showed a slight increase in the SH120 group relative to placebo (Fig. 7d and e). PCA, a less abundant but highly hygroscopic derivative of glutamate and a key NMF component, showed only minor changes from baseline and mirrored the general trend observed for filaggrin-derived components (Fig. 7f). However, in contrast to glycine and serine, its increase in the SH120 group reached statistical significance compared to placebo after three months and a similar trend was observed in the SH60 group approaching significance threshold (*p* = 0.087).

## Discussion

### Skin hydration

Maintaining adequate hydration of SC is fundamental for preserving skin barrier integrity and ensuring overall skin health and appearance. Insufficient hydration is frequently associated with clinical symptoms such as pruritus and the worsening of inflammatory skin conditions including atopic dermatitis and psoriasis^31^. Therefore, enhancing SC hydration through targeted interventions such as oral SH supplementation may offer both functional and health benefits.

Multiple clinical studies (Table 2) have demonstrated a positive effect of oral SH supplementation on skin hydration, with varying levels of statistical significance^4,8–16^. These effects have been reported across a wide range of Mw, including high Mw (800 kDa^11,14^, medium Mw (300 kDa^9,11,13^, and very low Mw (< 5 kDa^12^, with daily dosages ranging from 100 to 280 mg.

Our study showed a significant increase (difference versus placebo) in facial skin hydration on both the forehead and the cheek following oral administration of high-Mw SH (1.8 MDa) at both tested doses: 60 mg/day representing a low dosage not tested in any previous studies, and 120 mg/day representing a standard dose consistent with prior studies. The hydration trends differed between anatomical sites: while the expected seasonal decline was clearly visible on the forehead (and prevented by SH), cheek hydration did not decrease during the study period. Because the study ended in early December when winter had only just begun, the negative impact of cold and dry weather may not yet have manifested consistently across all measured sites, potentially contributing to the variability in hydration trends across anatomical sites. Nevertheless, this discrepancy does not diminish the validity of our findings, as the significant differences versus placebo remain the most relevant clinical outcomes in a superiority-designed RCT.

In case of the volar forearm, no consistent seasonal changes were observed, likely because this site is generally protected by clothing and less exposed to environmental stressors. No effect of SH was observed on this site. We speculate that SH may confer greater benefits at skin sites exposed to environmental challenge such as the face, consistent with proposed mechanisms of action (anti-inflammatory and anti-oxidative effects) as will be further discussed.

Hydration of SC is primarily maintained by hygroscopic molecules collectively referred to as NMF. NMF consists of various amino acids and their derivatives (urea, pyrrolidone carboxylic acid (PCA), glutamic acid, urocanic acid (UCA) etc.) derived primarily from filaggrin degradation, inorganic salts and lactate originating mainly from eccrine sweat^32,33^.

In this study, we found a slight increase in filaggrin-derived NMF components on the volar forearm, with PCA showing a statistically significant change. This may suggest a partial contribution of increased NMF to the enhanced facial hydration. On the other hand, the observed decrease in urea levels across all groups, irrespective of treatment, may reflect reduced sweat production due to seasonal factors, as the study ran from September to December. These findings highlight the complex and heterogeneous dynamics of individual NMF components and the limitations of our analysis, which did not include other key skin hydration contributors such as lactate, sugars, or electrolytes (e.g., Na⁺, K⁺). A more comprehensive analysis including other major NMF constituents and their regulators will be essential to clarify the mechanisms behind the observed changes in skin hydration.

Although facial skin hydration significantly increased following oral SH supplementation, participants did not subjectively perceive this improvement, as indicated by questionnaire responses. Self-assessed hydration levels and changes from baseline appeared strongly influenced by expectation or placebo effects. Moreover, the perception of skin hydration is very complex and influenced by factors such as sebum levels, skin texture, tightness, and irritation, rather than actual water content in SC. According to the Baumann Skin Type, “dry” skin is defined primarily by lipid deficiency, increased TEWL, and sensitivity rather than hydration levels^34^. Supporting this, a Korean study found that only sebum levels, not hydration, correlated with subjective classification of dry skin^35^.

**Table 2:** Summary of published clinical trials evaluating the effect of orally administered SH on facial skin hydration and other skin parameters (sorted in descending order by publication date). F female; Mw molecular weight; ND not determined; QA questionnaire assessment; SH sodium hyaluronate; y years.

Study, citation, location	*n*, % F, age	Skin type	SH daily dose, Mw, study duration	Hydration (SH vs. placebo)	Other effects (SH vs. placebo): ↑ sign. improvement, (↑) non-sign. improvement, - no difference
Nobile et al., 2025^8^, Italy and China	*n* = 42, 69% F, 34–65 y	Mild-to-moderate signs of skin aging (phototype II-VI)	200 mg, ND 8 weeks	+ 14.4% (*p* < 0.001)	$\uparrow$ Eyebags reduction ↑ Smoothness (Ra), roughness (Rz) ↑ Firmness (R0), elasticity (R2) ↑ Brightness ↑ Skin thickness ↑ Dermal thickness and density ↑ Wrinkle reduction $\uparrow$ Skin tone evenness (↑) Nasolabial folds reduction
Gao et al., 2023^9^, China	*n* = 129, 100% F, 18–65 y	Dry/oily/normal	100/200 mg, 300 kDa, 12 weeks	+ 6.9% (100 mg)* (n.s., *p* > 0.1)^#^ + 21.6% (200 mg)* (*p* < 0.01)^#^	(signif. SH vs. placebo not stated) (↑) Dermal density - Dermal, epidermal thickness - ITA°
Hsu et al., 2021^10^, China	*n* = 40, 72,5% F, 35–64 y	Normal	120 mg, ND, 12 weeks	+ 11.5% (*p* < 0.05)	↑ TEWL reduction ↑ Skin firmness (R0) ↑ Smoothness (SEsm, Var) (↑) Wrinkle reduction (SEw, Visia) (↑) Dermal thickness, intensity (↑) Scaliness reduction (SEsc) - Roughness (SEr) - Skin elasticity (R1, R2, R7)
Michelotti et al., 2021^4^, Italy	*n* = 60, 100% F, 35–70 y	Mild to moderate signs of aging	200 mg, Full spectrum, 4 weeks	+ 9.5% (*p* < 0.01)	↑ Wrinkles ↑ Elasticity (R2) (↑) TEWL (↑) Skin firmness (R0) (↑) Serum level of SH
Oe et al., 2017,^47^ Japan	*n* = 50, 60% F, 22–59 y	Presence of crow’s feet wrinkles	120 mg, 2/300 kDa 12 weeks	ND	↑ Wrinkle reduction - QA: luster, suppleness, wrinkles
Kawada et al., 2015^11^, Japan	*n* = 61, 100% F, 35–60 y	Dry, sagging, eye wrinkles	120 mg 800/300 kDa, 6 weeks	+ 28.1% (800 kDa) (n.s., *p* > 0.05) + 19.4% (300 kDa) (*p* < 0.05)	- Viscoelasticity ↑ QA: luster, suppleness (↑) QA: wrinkle reduction
Terashita et al., 2011^12^, China	*n* = 107, 84% F, 38–51 y	Dry	280 mg, Mix 1,5 + 5 kDa, 30 days	+ 26.1% (*p* < 0.01)	Increase in pH (*p* < 0.01)
Yoshida et al., 2009^13^, Japan	*n* = 42, 100% F, mean 43.3 y	Dry, rough, eye wrinkles, low elasticity	120 mg, 300 kDa, 6 weeks	+ 19.4% (*p* < 0.05)	- Wrinkles - Elasticity
Sato et al., 2007^14^, Japan	*n* = 39, 100% F, 37–59 y	Dry, rough, low elasticity, wrinkles	120 mg, 800 kDa, 6 weeks	+ 35.8% (n.s., *p* < 0.1)	- Wrinkle reduction - Elasticity - Roughness
Sato et al., 2002^15^, Japan	*n* = 35, 63% F, 18–45 y	Dry, rough	120 mg, ND, 4 weeks	−2.2%	- Smoothness (SEsm) - Roughness (SEr) - Scaliness (SEsc) - Wrinkles (SEw)
Kajimoto et al., 2001^16^, Japan	*n* = 22, 87% F, mean 26,7 y	Dry, rough	240 mg, ND, 6 weeks	+ 9.2% (n.s.)	- pH - Smoothness (SEsm) - Roughness (SEr) - Scaliness (SEsc) - Wrinkles (SEw)

*The results were calculated for all participants, irrespective of age group or skin type, based on raw data kindly provided by the authors.

^#^Calculated from raw data kindly provided by the authors (unpaired t-test).

### Skin barrier, TEWL, redness and sensitivity

Skin barrier function is mainly attributed to SC composed of corneocytes embedded in a matrix of highly organized intercellular lipids. Additionally, the viable layers of the epidermis contribute to this function through specialized cell-cell junctions (for a review, see e.g. Proksch et al.^36^. Disruption of any of these components can compromise the integrity of the skin barrier and is often associated with the development or exacerbation of various dermatological diseases, including atopic dermatitis. TEWL is a well-established parameter used to evaluate the barrier function of the skin, as it reflects the passive diffusion of water from the epidermis into the external environment^37^.

In the present study, oral supplementation with SH resulted in a significant reduction in TEWL on both the forehead and cheek at the end of the intervention period indicating an improvement in skin barrier function. Similar TEWL reductions following SH administration have been reported in previous studies (Table 2)^4,10^, although only Hsu et al.^10^ demonstrated a statistically significant difference between the SH and placebo group. Interestingly, their study reported a significant decrease in TEWL only after three months of supplementation, with no significant changes observed after one or two months, which is consistent with the temporal pattern observed in our study.

The underlying mechanisms of the observed TEWL reduction in our study remain incompletely understood. The slight, but significant increase in epidermal thickness observed in the SH120 group may suggest enhanced keratinocyte proliferation and/or terminal differentiation. This interpretation is further supported by the trend toward elevated levels of filaggrin-derived NMF, as filaggrin is a crucial protein of the epidermal differentiation complex and it also plays an essential role in the structural integrity and barrier function of SC^38^.

Enhanced skin barrier function may also be associated with the observed ability of orally administered SH to prevent the increase in facial redness that was seen in the placebo group, although this trend did not reach statistical significance. This rise in redness in the placebo group was likely driven by seasonal factors, particularly exposure to cooler and drier air typical for the transition from early autumn to early winter in Central Europe. Such environmental conditions are known to impair skin barrier integrity and increase skin reactivity and redness^39,40^. Indeed, a slightly increased skin sensitivity was reported by participants in the placebo group, while an opposite trend, a modest reduction in perceived sensitivity, was observed among volunteers receiving SH supplementation, providing additional support for the skin barrier-enhancing effects of SH.

In contrast to changes in facial redness areas, other colorimetric parameters associated with red color, including the erythema index and the a* value, did not differ between the SH and placebo groups. However, all groups exhibited a gradual decline in these values over the study period. This seasonal decline was accompanied by a reduction in the melanin index and an increase in skin lightness (ITA°), most likely reflecting the progressive fading of summer-induced tanning, which is in agreement with our previous study^7^.

Taken together, our findings suggest that oral SH administration may provide potential benefit from a health perspective for individuals with sensitive skin or compromised barrier function, as well as those suffering from dermatological conditions characterized by barrier dysfunction, including atopic dermatitis, psoriasis or rosacea.

### Sebum

Sebum is an oily substance secreted by sebaceous glands, composed primarily of triglycerides, free fatty acids, wax esters, squalene, cholesterol, and cellular debris^41^. On the face, a typical distribution pattern is observed, with higher sebum levels in the so-called T-zone (forehead, nose, chin) and lower levels on the cheeks^42^, which was confirmed by our data as well (Table S5; *p* < 0.001). Sebum fulfills several physiological functions, including lubrication of the skin and hair, contribution to thermoregulation, maintenance of the acid mantle important for skin barrier integrity, and modulation of the skin microbiome^43^.

Given its role in thermoregulation, sebum secretion typically decreases with declining environmental temperatures during colder months^7^. Unexpectedly, we observed a significant increase in sebum levels in the placebo group after three months at both the forehead and cheek, despite the fact that average outdoor temperatures during the study period showed the expected seasonal decrease (15.0 °C in September to 0.9 °C in December 2024; Czech Hydrometeorological Institute). The cause of this paradoxical rise remains unclear. Notably, this seasonal increase in sebum was significantly attenuated in both SH-supplemented groups, suggesting a possible effect of oral SH on sebum normalization.

Interestingly, although instrumentally measured sebum levels increased in the placebo group, subjective reports from participants indicated a perceived lower level of skin oiliness across all groups. This perceived decline was slightly more pronounced in the SH groups, potentially supporting the sebum-reducing effects of SH supplementation. While questionnaire-based outcomes are generally considered supplementary in this study, in the case of skin oiliness, previous research has demonstrated a strong correlation between subjective perception and instrumental measurements^35^, which adds weight to the relevance of these findings.

To the best of our knowledge, no previous study has specifically evaluated the effects of oral SH supplementation on facial sebum production. Our preliminary findings suggest that oral SH may have a normalizing or reducing effect on sebum levels, which could be particularly relevant for individuals with hyperseborrhea and related skin conditions such as acne and seborrheic dermatitis. However, given the unexpected pattern in the placebo group and the lack of mechanistic understanding, these results should be interpreted with caution and confirmed in further studies.

### Skin elasticity, wrinkles and collagen level

Skin biomechanical properties are primarily determined by the composition and organization of the dermal extracellular matrix, including fibrillar collagens (types I and III) which confer tensile strength and stiffness, elastic fibers responsible for recoil after deformation, and the viscoelastic ground substance rich in molecules such as hyaluronan. The loss of skin elasticity is typically associated with the development of wrinkles; together, they represent key hallmarks of cutaneous ageing^44^.

In this study, we observed a simultaneous decline in parameters associated with skin recovery (R2, R5, R7), deformation (R0, R1, R3, R4), and viscosity (R6) over the course of the study. This pattern suggests that the skin became less elastic, firmer, and less distensible, likely due to seasonal influences. A comparable seasonal decline in elasticity-related parameters (particularly R2, R5, and R7) has also been reported in a previous study, although with a smaller amplitude than observed here^45^. While the magnitude of the reduction in R-values in our study was striking, the decline was observed consistently across all study groups, across all independent R parameters, and followed gradual temporal trends. This supports the interpretation that the reduction reflected genuine seasonal changes in skin mechanical properties rather than measurement errors or device malfunction.

Importantly, the comparison versus placebo, which is central to a superiority-designed RCT, showed that supplementation with SH had a modest positive effect on these changes. Specifically, the reduction in R2, R5, and R7 was less pronounced indicating a preservation of skin elasticity, while the continued decrease in R0, R1, R3, and R4 suggested enhanced skin firmness. Additionally, a slight increase in R6 (viscosity) might indirectly reflect improved hydration status. Prior studies have also documented improvements in skin elasticity following SH supplementation, most frequently by increase in R2 values (Table 2)^4,8,10^.

Similarly, dermal density reflecting the collagen content within the dermis^46^ also decreased during the study which could contribute to the elasticity decline. SH supplementation mitigated this decline, in line with findings from other studies showing increased dermal density following SH intake (Table 2)^8–10^.

Regarding wrinkles, a significant reduction in periorbital wrinkles was observed in both SH groups compared to placebo, consistent with findings from previous clinical trials (Table 2)^4,8,10,11,47^. A minor seasonal improvement was also detected in the placebo group. This finding is somewhat unexpected, as wrinkle reduction is typically associated with improved skin elasticity and increased collagen content, yet both parameters declined in the placebo group. Therefore, additional seasonal factors must have contributed to the observed wrinkle improvement, such as enhanced skin hydration or reduced photodamage, although the underlying mechanisms remain unclear. In contrast, SH supplementation produced a more coherent and physiologically plausible outcome, where wrinkle reduction was accompanied by preservation or enhancement of both skin elasticity and dermal density.

### Mechanisms of orally administered SH action

#### SH metabolism and bioavailability

While early studies based on radioactive isotope-labeling suggested that orally administered SH could be absorbed in the gastrointestinal tract, transported to peripheral tissues such as skin and joints, and exert direct local effects^48,49^ other studies have suggested that this scenario is rather unlikely^50–52^.

Our recent investigation^51^ employing orally administered 13C-labeled SH in mice, combined with advanced LC-MS analysis, demonstrated that high-Mw SH (1.6 MDa) undergoes partial depolymerization in the stomach to fragments of ~150–600 kDa due to acidic hydrolysis and microbial degradation, but the vast majority (~97% of the administered dose) was degraded by gut microbiota into unsaturated oligosaccharides, primarily disaccharides. These low-Mw fragments were shown to enter systemic circulation, albeit at very low levels (~0.2%), irrespective of the initial Mw of the administered SH. Though a certain limited absorption of unsaturated oligosaccharides was found, the major part was detected in urine and plasma, while their concentrations in peripheral tissues such as skin or joints remained below the detection limit suggesting that a direct action of SH and its metabolites in peripheral tissues, including the skin, is rather improbable^51^.

These findings indicate that the effects of orally administered SH are more plausibly mediated through complex systemic regulatory mechanisms rather than through direct deposition or activity of SH and/or its metabolites in distal tissues, including the skin.

#### Possible regulatory mechanisms of orally administered SH

Orally administered SH may exert systemic and skin-related effects through several interconnected mechanisms.

Modulation of the gut microbiota is one of the frequently discussed mechanisms. SH has been shown to promote the growth of short-chain fatty acid (SCFA)-producing bacteria and xylan/cellulose-degrading species, including *Bacteroides* and *Bifidobacterium* spp., in mouse models^51,53^. Indeed, increased levels of propionate and butyrate were observed in SH-supplemented animals^51^. These SCFAs are known to exhibit multiple systemic and skin-related benefits, including anti-inflammatory effects, promotion of keratinocyte differentiation, and enhancement of the skin barrier function^54^.

Another proposed mechanism involves direct interaction of SH or its fragments with hyaluronan-binding receptors in the gut epithelium, such as CD44, toll-like receptor 4 (TLR4), and layilin, which may mediate intestinal epithelial barrier enhancement and anti-inflammatory effects (for a review, see Kim et de la Motte^55^). Generally, anti-inflammatory activity is widely considered a central mechanism of SH´s beneficial action. For instance, in a Th1-type autoimmune mouse model, orally administered SH (900 kDa) was shown to bind to TLR4 on colonic epithelial cells, resulting in increased expression of the anti-inflammatory cytokine IL-10 and decreased levels of pro-inflammatory mediators^50^. Similarly, in a UV-induced photodamage model, oral SH reduced pro-inflammatory IL-1β and IL-6 expression in the skin^56^. In a comparable UV-exposed mouse model treated with SH (1.73 MDa), we also demonstrated attenuation of UV-induced increases in pro-inflammatory prostaglandins PGD₂ and PGE₂ in the skin^57^.

The anti-inflammatory action of SH may be partly mediated by a reduction of oxidative stress. A study found increased levels of the antioxidant enzyme superoxide dismutase in muscle and liver tissue, along with decreased serum concentrations of malondialdehyde (MDA), a marker of lipid peroxidation and oxidative stress, in mice fed with SH^53^. Our own findings similarly showed reduced MDA in liver following SH administration in a mouse model^57^.

Furthermore, SH supplementation has been associated with increased collagen levels in the skin. For instance, in photodamaged mouse skin, SH treatment led to elevated levels of collagen-stimulating TGF-β and downregulation of collagen-degrading matrix metalloproteinase 1 (MMP1) which were accompanied by an overall increase in collagen levels^56^. Our data also confirmed increased TGF-β and collagen I gene expression, and downregulation of MMP9 in the UV-damaged mouse skin, together with increased plasma levels of α-ketoglutarate, a known collagen synthesis cofactor, following SH supplementation^57^. Additionally, an increase in hyaluronan synthase 2 (*HAS2*) gene expression and dermal hyaluronan level was found.

Together, these mechanisms, including antioxidant and anti-inflammatory activity, enhanced keratinocyte differentiation, and upregulation of collagen and hyaluronan synthesis, might have contributed to the attenuation of seasonal skin deterioration, such as decreased elasticity, collagen loss, and increased facial redness observed in this clinical study. They could also play a role in increased skin hydration, improved barrier function, and wrinkle reduction.

Interestingly, SH (50 kDa) was also shown to affect lipid metabolism and exert anti-obesity effect by inhibiting adipogenesis in mice on a high-fat diet and downregulating key adipogenic transcription factors, including PPAR-γ and SREBP-c^58,59^. Because adipocytes and sebocytes share several functional and molecular characteristics, including lipogenic differentiation, lipid accumulation, and responsiveness to similar regulatory stimuli^60^, it is plausible that the reduction in sebum production observed in our study may reflect the systemic anti-lipogenic activity of SH.

## Conclusions

This randomized, placebo-controlled clinical trial provides robust evidence that oral supplementation with high-Mw SH (1.8 MDa) at daily doses of 60 mg and 120 mg improves multiple objective and subjective parameters of skin condition in healthy adult Caucasian subjects. A dose-dependent trend was observed across several outcomes, with the higher 120 mg dose producing more pronounced and consistent effects. However, confirmation of statistically significant dose-response relationships would require a larger sample size.

Although the precise mechanisms of action remain to be fully elucidated, evidence from this and previous preclinical studies supports a multifactorial mode of action involving antioxidant and anti-inflammatory activity, remodeling of dermal matrix, modulation of NMF composition and lipid metabolism.

Taken together, these findings support the potential of oral SH as a safe and effective nutritional intervention for maintaining or improving skin health and appearance.

This trial is limited by its relatively short duration, potential seasonal confounders, and limited mechanistic resolution (e.g., incomplete NMF profile). The study population consisted of healthy Caucasian adults with Fitzpatrick skin types I–III, which may limit generalizability to more diverse skin types and ethnic groups. While objective assessments were comprehensive, mechanistic interpretations remain largely hypothetical and based on preclinical animal data.

## Supplementary Information

Below is the link to the electronic supplementary material.

Supplementary Material 1

## Data Availability

Aggregated datasets (raw values and values expressed as % of baseline) are provided in the Supplementary material. De-identified individual participant data are available from the corresponding author (iva.doleckova@contipro.com) upon reasonable request.

## Abbreviations

EFSA: The European Food Safety Authority
MDA: Malondialdehyde
MMP: Matrix Metalloproteinase
NMF: Natural Moisturizing Factor
PCA: Pyrrolidone Carboxylic Acid
RCT: Randomized Controlled Trial
SC: Stratum Corneum
SCFA: Short-chain Fatty Acid
SH: Sodium Hyaluronate
SH60: Sodium Hyaluronate, daily dose 60 mg
SH120: Sodium Hyaluronate, daily dose 120 mg
SPIRIT: Standard Protocol Items: Recommendations for Interventional Trials 2013
TEWL: Transepidermal Water Loss
TLR4: Toll-like Receptor 4
