# Supplementary material for: Oral sodium hyaluronate improves skin hydration, barrier function and signs of aging: a randomized, double-blind, placebo-controlled trial in 150 healthy adults
Source: Sci Rep. 2025 Dec 20;16:2941. doi: 10.1038/s41598-025-32758-5 (PMC12827323; doi:10.1038/s41598-025-32758-5)
Supplement: Supplementary file 1 — Supplementary Material 1 [file 41598_2025_32758_MOESM1_ESM.pdf]

SUPPLEMENTARY MATERIAL

Details about the composition and microbial safety of the SH60, SH120 and placebo solutions.

Composition of SH60, SH120 and placebo solutions is shown in **Table S1**. SH (Nutrihyl®, grade suitable for use in food supplements) used in this study was of microbial origin (*Streptococcus equi* subs. *zooepidemicus*), manufactured by Contipro a.s. (Czech Rep.) and had molecular weight (Mw) of 1.8 MDa. Citric acid (monohydrate) was provided from Jiangsu Guoxin Union Energy (China), potassium sorbate and sodium benzoate from Hages (Czech Rep.), xantham gum from Fichema (Czech Rep.), and orange flavor (SC1073891) from Tastepoint (Slovenia), all ingredients were in food grade. The solutions were manufactured in a facility designated for the production of dietary supplements. Microbiological safety was assured by determining the total count of bacteria (≤ 1 CFU/mL, all three solutions), molds, yeasts, and coliform bacteria (all < 1 CFU/mL, all three solutions). The efficacy of the product's preservation was verified by a challenge test (based on Ph.Eur. Efficacy of antimicrobial preservation).

The chemical stability of SH in the solution was supported by internal data from Contipro a.s., demonstrating that aqueous SH solutions remain stable for at least 24 months at 25 °C under standard conditions, and for 6 months at 40 °C under accelerated conditions (data not shown). Although no formal stability testing was conducted on the final SH60 and SH120 formulations used in this study, all tested products were stored at 4–8 °C throughout the study period to ensure maximal preservation of quality.

Table S1. Composition of the SH60, SH120 and placebo solutions.

**Ingredient**	**g/L**		
	**SH60**	**SH120**	**Placebo**
Water	to 1000 mL	to 1000 mL	to 1000 mL
Sugar	45.0	45.0	45.0
Citric acid	2.4	2.4	2.4
Potassium sorbate	1.0	1.0	1.0
Sodium benzoate	1.0	1.0	1.0
**Sodium hyaluronate**	**4.0**	**8.0**	**0.0**
**Xanthan gum**	**3.5**	**0.0**	**7.0**
Orange flavor	1.4	1.4	1.4

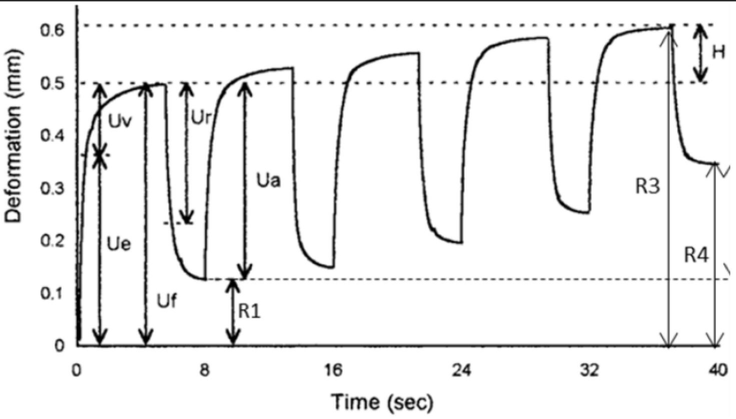

Param.	Description	Physiological meaning	Interpretation
**R0**	Maximum amplitude (Uf)	Pliability/firmness of the skin. Amplitude at the end of the suction phase of the first cycle.	Lower = firmer and less distensible skin.
**R1**	Immediate retraction (Uf – Ua)	Ability of the skin to recover its initial state after first deformation (residual deformation in mm at the end of the first recovery phase)	Lower = better elastic recoil.
**R2**	Overall/gross elasticity (Ua/Uf)	Reflects gross elasticity (elastic + viscous recovery).	Higher = better elasticity (closer to 1 = better elasticity).
**R3**	Last maximum amplitude	Reflects fatigue after repeated suction.	Lower = lower tiring effect, fatigue or structural weakness.
**R4**	Last minimum amplitude	Ability of the skin to recover its initial state after repeated deformation (residual deformation in mm at the end of the last recovery phase)	Lower = better elastic recovery.
**R5**	Net elasticity (Ur / Ue)	Measures pure elastic recovery without viscous components.	Higher = better elasticity.
**R6**	Viscoelastic ratio (Uv / Ue)	Reflects viscous deformation.	Lower = more elastic than viscous response.
**R7**	Biological elasticity (Ur / Uf)	Indicates the skin's ability to return to its original position.	Higher = better biological elasticity.
**R8**	Total recovery (Ua)	Shows extent of recovery regardless of deformation amplitude.	Higher = better total recovery.
**R9**	Fatigue ratio (H, R3 – R0)	Measures difference in maximum deformation over time, representing skin fatigue.	Lower = less fatigue.

Figure S1. Illustration of the Cutometer® deformation-time curve and overview of derived biomechanical skin parameters R0–R9 with their description.

Questionnaire for the participants

Name	
Code of supplement	
Filling date	
Study time point<br>(0, 2, 4, 6, 8, 10, 12 weeks)	
Weight of supplement incl. bottle (g) (filled by investigator from Contipro)	

Number of missed doses in the last 2 weeks	
Observed negative side effects	
Observed other unintended effects	
Events in the last 2 weeks which could have an impact on the skin (face, forearm) or gastrointestinal system (diseases, accidents, longer stay in significantly different environment (exotic holiday etc.), excessive sunbathing etc.)	

Subjective assessment of the facial skin parameters (mark on the scale 1-5):

**Hydration**	Hydrated	1	2	3	4	5	Dry
**Roughness**	Smooth	1	2	3	4	5	Rough
**Oiliness**	Dry	1	2	3	4	5	Oily
**Elasticity**	Good<br>(skin is tight, after deformation it returns fast to the original state)	1	2	3	4	5	Low<br>(skin is loose, after deformation it returns very slowly to the original state)
**Eye wrinkles – number**	None	1	2	3	4	5	Many
**Eye wrinkles – depth**	None	1	2	3	4	5	Very deep
**Skin sensitivity**	Low<br>(never get irritated, no burning, itching sensations)	1	2	3	4	5	High<br>(frequently get irritated, common burning, itching sensations)

Changes in the skin parameters from baseline:

**Hydration**	Significantly<br>more hydrated	Slightly<br>more hydrated	No changes	Slightly<br>drier	Significantly<br>drier
**Roughness**	Significantly<br>smoother	Slightly<br>smoother	No changes	Slightly<br>rougher	Significantly<br>rougher
**Oiliness**	Significantly<br>less oily	Slightly<br>less oily	No changes	Slightly<br>more oily	Significantly<br>more oily
**Elasticity**	Significantly<br>better	Slightly<br>better	No changes	Slightly<br>worse	Significantly<br>worse
**Eye wrinkles – number**	Significantly<br>fewer	Slightly<br>fewer	No changes	Slightly<br>more	Significantly<br>more
**Eye wrinkles – depth**	Significantly<br>smaller	Slightly<br>smaller	No changes	Slightly<br>deeper	Significantly<br>deeper
**Skin sensitivity**	Significantly<br>less sensitive	Slightly<br>less sensitive	No changes	Slightly<br>more sensitive	Significantly<br>more sensitive

Figure S2. Questionnaire for the participants translated to English.

Table S2. Mean number of reported missed doses and average daily intake (mL). Values are presented as mean ± SD.

	Placebo	SH60	SH120
Missed doses per participant	1.86 ± 2.76	2.12 ± 3.29	1.52 ± 1.80
Daily intake (mL)	14.2 ± 2.1	13.9 ± 1.9	14.5 ± 1.5

Table S3. Adverse events reported during the study.

	Placebo (month)						SH60 (month)						SH120 (month)					
	0,5	1	1,5	2	2,5	3	0,5	1	1,5	2	2,5	3	0,5	1	1,5	2	2,5	3
Acne, pimples, pustules		2				1		2			1	2	1	1	1	2	1	1
Increased scalp oiliness / greasier hair						1												
Itching of the scalp or face		1							1					1		1		
Joint pain				1		1												
Dry skin on legs or body						1						1						
Hair loss / increased hair shedding								1	1	1	1	1	1					
Dry lips							1	1		1								
Dry skin		1																
Overall worsening of skin condition							1											
Increased skin sensitivity								1										
Transient bloating									1									

Table S4. Other unintended effects reported during the study.

	Placebo (month)						SH60 (month)						SH120 (month)					
	0,5	1	1,5	2	2,5	3	0,5	1	1,5	2	2,5	3	0,5	1	1,5	2	2,5	3
Hair improvement (faster growth, reduced hair loss, less oiliness and breakage)	1	2	5	5	5	8		1	2	2	4	4	1	4	4	2	6	5
Stronger or healthier nails		2	5	5	8	7		1	3	3	2	5	2	2	3	3	6	5
Improved joint function, reduced musculoskeletal discomfort	1	2	2	5	3	2	1	1	2		1		3	4		1	2	1
Skin improvement	2	2	1	5		3	1	2	1	3	2	5	2	1		5	3	8
Improvement in urinary tract inflammation	1	1																
Improvement in acne or inflamed hair follicles				1														
Reduction of swelling (e.g., elbow)			1	1	1	1												
Enhanced eyelash appearance or growth				1	2	1												
Improved digestive or intestinal function						1												
Reduced nail fold inflammation or hangnails							1	1										
Improved wound healing or skin recovery																		
Fuller and more hydrated lips									1				1					
Reduction in under-eye puffiness or swelling										1								
Improved visual comfort and reduced eye fatigue												1						
Reduction in acne symptoms																1	1	

Table S5. Complete aggregated datasets for all instrumental-based skin parameters, including raw values and percentage of baseline (%T0).

HYDRATION							
Site	Time point	A.U. mean ± SD (95% CI)			%T0 mean ± SD (95% CI)		
		Placebo	SH60	SH120	Placebo	SH60	SH120
Forehead	T0	55 ± 9.4 (52.3–57.7)	58.2 ± 8.8 (55.7–60.7)	56.1 ± 9.8 (53.3–58.9)	100 ± 0 (100–100)	100 ± 0 (100–100)	100 ± 0 (100–100)
	M1	51.7 ± 11.1 (48.5–54.8)	55.7 ± 11.7 (52.3–59.1)	51 ± 10.8 (47.9–54.1)	95 ± 18.3 (89.8–100.2)	96.1 ± 17.1 (91.2–101)	92.3 ± 19.1 (86.8–97.7)
	M2	53 ± 8.7 (50.5–55.4)	55.5 ± 11.1 (52.3–58.8)	54.1 ± 12.1 (50.7–57.6)	98.3 ± 19.1 (92.8–103.7)	95.8 ± 16.1 (91.2–100.5)	97.5 ± 19.3 (92.1–103)
	M3	50 ± 10.2 (47.1–52.9)	58.3 ± 10 (55.3–61.2)	55.3 ± 13.4 (51.5–59.1)	92.5 ± 21.3 (86.4–98.5)	101.2 ± 14.1 (97.1–105.4)	99.7 ± 21 (93.7–105.6)
Cheek	T0	53 ± 10.2 (50.1–55.9)	52.7 ± 9.2 (50.1–55.3)	52.6 ± 11.7 (49.3–56)	100 ± 0 (100–100)	100 ± 0 (100–100)	100 ± 0 (100–100)
	M1	51.4 ± 11.1 (48.2–54.6)	52.2 ± 10.9 (49.1–55.4)	50.3 ± 11 (47.2–53.5)	97.8 ± 17.8 (92.8–102.9)	100.5 ± 21.2 (94.4–106.6)	98.6 ± 23.7 (91.9–105.3)
	M2	55 ± 11.9 (51.6–58.4)	55.1 ± 10.9 (51.9–58.3)	56.5 ± 12.5 (52.9–60)	105.1 ± 22.1 (98.8–111.4)	106.7 ± 23.7 (99.9–113.6)	109.7 ± 22.7 (103.3–116.1)
	M3	56.2 ± 12.5 (52.7–59.8)	60.1 ± 11.5 (56.8–63.5)	60.9 ± 13.2 (57.2–64.6)	107.3 ± 21.2 (101.3–113.4)	116.4 ± 20.6 (110.3–122.5)	118.9 ± 27.3 (111.1–126.6)
Forearm	T0	42.8 ± 6.8 (40.9–44.7)	44.4 ± 9 (41.8–47)	43.2 ± 9.1 (40.7–45.8)	100 ± 0 (100–100)	100 ± 0 (100–100)	100 ± 0 (100–100)
	M1	41.7 ± 7.9 (39.4–43.9)	40.3 ± 8.9 (37.7–42.9)	41.2 ± 7.4 (39.1–43.4)	98.8 ± 19.5 (93.2–104.3)	91.7 ± 15.1 (87.4–96.1)	97.7 ± 19.2 (92.2–103.2)
	M2	41.8 ± 7.6 (39.6–44)	43.6 ± 8 (41.2–45.9)	42.9 ± 6.6 (41–44.7)	99 ± 17.3 (94.1–103.9)	100.7 ± 19.4 (95–106.3)	101.6 ± 18.1 (96.5–106.7)
	M3	44.6 ± 6.9 (42.6–46.6)	42.9 ± 7 (40.8–44.9)	43.9 ± 7.3 (41.8–45.9)	106.2 ± 20.4 (100.4–112)	99.6 ± 18.8 (94.1–105.1)	104.2 ± 20.3 (98.4–109.9)
TEWL							
Site	Time point	$g/m^2/h$ mean ± SD (95% CI)			%T0 mean ± SD (95% CI)		
		Placebo	SH60	SH120	Placebo	SH60	SH120
Forehead	T0	19.1 ± 3.5 (18.1–20.1)	20 ± 5 (18.5–21.4)	19.2 ± 4.1 (18–20.4)	100 ± 0 (100–100)	100 ± 0 (100–100)	100 ± 0 (100–100)
	M1	19.6 ± 4.4 (18.4–20.9)	21.2 ± 7.2 (19.1–23.3)	19.1 ± 3.8 (18–20.1)	104.4 ± 22.4 (98.1–110.8)	106 ± 17.7 (100.9–111.1)	101.1 ± 18.4 (95.9–106.3)
	M2	18.7 ± 4.2 (17.6–19.9)	20 ± 4.1 (18.8–21.2)	19.1 ± 4 (18–20.3)	100.2 ± 23.3 (93.6–106.8)	103.4 ± 22.6 (96.8–110)	101.8 ± 21.1 (95.7–107.8)
	M3	18.3 ± 3.5 (17.3–19.3)	17.2 ± 5.1 (15.7–18.7)	16.1 ± 5 (14.7–17.6)	97.7 ± 19.1 (92.3–103.1)	88.3 ± 24.4 (81.1–95.4)	85.1 ± 24.1 (78.2–91.9)
Cheek	T0	19.1 ± 4 (18–20.3)	20.1 ± 5.8 (18.4–21.8)	19.7 ± 5.8 (18–21.3)	100 ± 0 (100–100)	100 ± 0 (100–100)	100 ± 0 (100–100)
	M1	19.2 ± 4.9 (17.8–20.6)	21.1 ± 6.9 (19.1–23)	19.8 ± 5.6 (18.2–21.4)	101.9 ± 22.4 (95.5–108.2)	106 ± 23.5 (99.2–112.7)	104.4 ± 27.3 (96.6–112.1)
	M2	18.5 ± 4.4 (17.3–19.7)	20.1 ± 4.8 (18.7–21.5)	19.6 ± 5.2 (18.1–21.1)	98.8 ± 24.2 (91.9–105.7)	103.6 ± 22.6 (97–110.1)	103.7 ± 29.7 (95.2–112.1)
	M3	18.9 ± 4.9 (17.5–20.3)	17.6 ± 5.3 (16.1–19.2)	16 ± 5.8 (14.4–17.7)	100.5 ± 24 (93.7–107.3)	89.9 ± 23.9 (82.9–97)	85.1 ± 29.4 (76.8–93.5)
SEBUM							
Site	Time point	$\mu g/cm^2$ mean ± SD (95% CI)			%T0 mean ± SD (95% CI)		
		Placebo	SH60	SH120	Placebo	SH60	SH120
Forehead	T0	99.1 ± 46 (86–112.2)	96.2 ± 38.7 (85.1–107.4)	102.4 ± 41.6 (90.5–114.2)	100 ± 0 (100–100)	100 ± 0 (100–100)	100 ± 0 (100–100)
	M1	95.4 ± 39.6 (84.1–106.6)	100.5 ± 44.5 (87.8–113.3)	103.7 ± 48.3 (90–117.4)	105 ± 37.3 (94.5–115.6)	112.3 ± 51 (97.7–126.9)	103.5 ± 30.2 (94.9–112.1)
	M2	95.3 ± 42.3 (83.3–107.3)	98.4 ± 44.9 (85.4–111.5)	94.9 ± 43.7 (82.5–107.3)	105.2 ± 42.1 (93.3–117.2)	109.7 ± 43.8 (97–122.5)	97.6 ± 35.5 (87.5–107.7)
	M3	108.7 ± 44 (96.2–121.2)	107.2 ± 40.2 (95.4–119)	104.9 ± 39.4 (93.7–116.1)	123.5 ± 53.4 (108.3–138.7)	129.1 ± 60.7 (111.3–146.9)	106.8 ± 29.3 (98.5–115.1)
Cheek	T0	66.4 ± 40.7 (54.9–78)	68.3 ± 31.2 (59.3–77.2)	77.2 ± 46.4 (64–90.4)	100 ± 0 (100–100)	100 ± 0 (100–100)	100 ± 0 (100–100)
	M1	59.3 ± 32.9 (49.9–68.6)	67.1 ± 33.7 (57.4–76.8)	70.8 ± 47 (57.5–84.2)	99.4 ± 42.5 (87.3–111.5)	105.1 ± 41 (93.3–116.9)	93.9 ± 33.3 (84.4–103.3)
	M2	59.9 ± 31.6 (51–68.9)	66.9 ± 32.5 (57.5–76.4)	62.8 ± 36.5 (52.4–73.1)	106.9 ± 63.8 (88.8–125)	106.6 ± 43.5 (94–119.3)	85.9 ± 30.2 (77.3–94.4)
	M3	70.8 ± 28.3 (62.7–78.8)	70.8 ± 30.2 (61.9–79.7)	73.6 ± 40.7 (62.1–85.2)	142.1 ± 112.3 (110.1–174)	117.5 ± 48.5 (103.3–131.8)	102.5 ± 35.5 (92.4–112.6)
WRINKLE DEPTH							
Site	Time point	µm			%T0 mean ± SD (95% CI)		
		Placebo	SH60	SH120	Placebo	SH60	SH120
Crow's feet	T0	182.9 ± 79.2 (160.4–205.5)	171.8 ± 74.7 (150.3–193.2)	185.1 ± 67.4 (165.9–204.2)	100 ± 0 (100–100)	100 ± 0 (100–100)	100 ± 0 (100–100)

	**M1**	170.7 ± 77.9<br>(148.6–192.9)	147.2 ± 71.5<br>(126.7–167.8)	153.3 ± 67.8<br>(134.1–172.6)	96.5 ± 19<br>(91.1–101.9)	86.6 ± 13.7<br>(82.6–90.5)	83.9 ± 21.5<br>(77.8–90)
	**M2**	159.9 ± 77.8<br>(137.8–182)	133.6 ± 58.3<br>(116.7–150.5)	134.8 ± 55.1<br>(119.2–150.5)	90.5 ± 25.1<br>(83.4–97.7)	79.1 ± 15.7<br>(74.5–83.6)	72.7 ± 14.6<br>(68.5–76.8)
	**M3**	156.1 ± 80.7<br>(133.1–179)	128.2 ± 60.2<br>(110.5–145.8)	127 ± 53.1<br>(111.9–142.1)	87.4 ± 24.8<br>(80.4–94.4)	75.8 ± 17.1<br>(70.8–80.9)	69.2 ± 16<br>(64.7–73.8)
**DERMAL DENSITY (COLLAGEN LEVEL)**							
**Site**	**Time point**	**%**			**%T0<br>mean ± SD (95% CI)**		
		**Placebo**	**SH60**	**SH120**	**Placebo**	**SH60**	**SH120**
**Forehead**	**T0**	6.8 ± 2.1<br>(6.2–7.4)	6.8 ± 2.2<br>(6.1–7.4)	6.3 ± 1.9<br>(5.7–6.8)	100 ± 0<br>(100–100)	100 ± 0<br>(100–100)	100 ± 0<br>(100–100)
	**M1**	6.1 ± 2<br>(5.5–6.6)	5.9 ± 1.9<br>(5.3–6.4)	6 ± 1.8<br>(5.5–6.6)	92.9 ± 34.5<br>(83.1–102.7)	91.3 ± 25.7<br>(83.9–98.7)	99.5 ± 27.4<br>(91.7–107.2)
	**M2**	5.9 ± 2.3<br>(5.2–6.5)	5.7 ± 1.8<br>(5.2–6.3)	6.1 ± 1.8<br>(5.6–6.6)	87.3 ± 25.9<br>(80–94.7)	90.5 ± 29.6<br>(81.9–99.1)	101.7 ± 30.5<br>(93–110.3)
	**M3**	5.7 ± 1.9<br>(5.1–6.2)	5.5 ± 1.6<br>(5.0–6.0)	5.7 ± 1.5<br>(5.2–6.1)	85 ± 24.4<br>(78.1–91.9)	87.2 ± 27.3<br>(79.3–95.2)	95 ± 29.1<br>(86.7–103.2)
**EPIDERMAL THICKNESS**							
**Site**	**Time point**	**μm**			**%T0<br>mean ± SD (95% CI)**		
		**Placebo**	**SH60**	**SH120**	**Placebo**	**SH60**	**SH120**
**Forehead**	**T0**	128.1 ± 15.6<br>(123.7–132.5)	126.9 ± 13.1<br>(123.1–130.7)	124 ± 14.5<br>(119.9–128.2)	100 ± 0<br>(100–100)	100 ± 0<br>(100–100)	100 ± 0<br>(100–100)
	**M1**	121.7 ± 12.9<br>(118.1–125.4)	121.8 ± 11.5<br>(118.5–125.1)	120.5 ± 12.3<br>(117–124)	94.7 ± 12.2<br>(91.2–98.2)	96.7 ± 11.2<br>(93.5–99.9)	98.1 ± 12.8<br>(94.5–101.7)
	**M2**	123 ± 12.8<br>(119.4–126.7)	121.4 ± 14.6<br>(117.2–125.7)	119.3 ± 11.5<br>(116–122.5)	95.9 ± 11.6<br>(92.6–99.3)	96.3 ± 12.1<br>(92.8–99.8)	97 ± 11.8<br>(93.7–100.3)
	**M3**	118.3 ± 11<br>(115.2–121.5)	116.4 ± 11.6<br>(113–119.8)	118.7 ± 10.2<br>(115.8–121.6)	92.1 ± 9.9<br>(89.2–94.9)	91.8 ± 10.3<br>(88.8–94.8)	96.7 ± 12.1<br>(93.3–100.1)
**ELASTICITY – R0**							
**Site**	**Time point**	**mm**			**%T0<br>mean ± SD (95% CI)**		
		**Placebo**	**SH60**	**SH120**	**Placebo**	**SH60**	**SH120**
**Forehead**	**T0**	0.034 ± 0.017<br>(0.029–0.038)	0.038 ± 0.021<br>(0.032–0.044)	0.034 ± 0.017<br>(0.029–0.039)	100 ± 0<br>(100–100)	100 ± 0<br>(100–100)	100 ± 0<br>(100–100)
	**M1**	0.03 ± 0.016<br>(0.025–0.034)	0.03 ± 0.016<br>(0.025–0.034)	0.03 ± 0.017<br>(0.026–0.035)	99.2 ± 51.4<br>(84.6–113.8)	89.9 ± 48.8<br>(75.9–103.9)	94.6 ± 39.6<br>(83.4–105.9)
	**M2**	0.028 ± 0.015<br>(0.024–0.032)	0.024 ± 0.011<br>(0.021–0.028)	0.026 ± 0.015<br>(0.021–0.03)	95 ± 47<br>(81.6–108.4)	79.1 ± 37.6<br>(68.2–90)	84 ± 43.5<br>(71.6–96.3)
	**M3**	0.021 ± 0.012<br>(0.017–0.024)	0.019 ± 0.011<br>(0.015–0.022)	0.015 ± 0.007<br>(0.013–0.017)	73.6 ± 47.6<br>(60.1–87.1)	59.6 ± 45.4<br>(46.3–72.9)	53.4 ± 28.6<br>(45.3–61.6)
**Cheek**	**T0**	0.046 ± 0.017<br>(0.041–0.051)	0.049 ± 0.017<br>(0.044–0.054)	0.047 ± 0.023<br>(0.04–0.053)	100 ± 0<br>(100–100)	100 ± 0<br>(100–100)	100 ± 0<br>(100–100)
	**M1**	0.039 ± 0.019<br>(0.034–0.044)	0.036 ± 0.017<br>(0.031–0.041)	0.038 ± 0.02<br>(0.032–0.044)	88.9 ± 35.1<br>(79–98.9)	76.1 ± 26.1<br>(68.6–83.6)	86.2 ± 33<br>(76.8–95.6)
	**M2**	0.03 ± 0.01<br>(0.027–0.033)	0.032 ± 0.011<br>(0.028–0.035)	0.028 ± 0.01<br>(0.025–0.031)	72.2 ± 31.4<br>(63.3–81.2)	70.5 ± 25.5<br>(63.1–77.9)	70 ± 31.2<br>(61.1–78.9)
	**M3**	0.022 ± 0.015<br>(0.018–0.026)	0.02 ± 0.007<br>(0.018–0.022)	0.02 ± 0.01<br>(0.017–0.023)	56.1 ± 47.1<br>(42.7–69.5)	46.9 ± 23.9<br>(39.9–54)	51 ± 29.5<br>(42.7–59.4)
**ELASTICITY – R1**							
**Site**	**Time point**	**mm**			**%T0<br>mean ± SD (95% CI)**		
		**Placebo**	**SH60**	**SH120**	**Placebo**	**SH60**	**SH120**
**Forehead**	**T0**	0.016 ± 0.009<br>(0.014–0.018)	0.018 ± 0.011<br>(0.015–0.021)	0.015 ± 0.008<br>(0.013–0.018)	100 ± 0<br>(100–100)	100 ± 0<br>(100–100)	100 ± 0<br>(100–100)
	**M1**	0.016 ± 0.008<br>(0.014–0.018)	0.015 ± 0.008<br>(0.013–0.018)	0.016 ± 0.008<br>(0.014–0.018)	119.1 ± 77<br>(97.2–140.9)	106.3 ± 87.8<br>(81.1–131.5)	118.1 ± 57.3<br>(101.8–134.4)
	**M2**	0.018 ± 0.011<br>(0.015–0.021)	0.016 ± 0.009<br>(0.013–0.018)	0.017 ± 0.013<br>(0.013–0.02)	129.1 ± 71.5<br>(108.8–149.5)	108 ± 61<br>(90.3–125.7)	117.4 ± 70.5<br>(97.4–137.4)
	**M3**	0.013 ± 0.009<br>(0.011–0.016)	0.012 ± 0.009<br>(0.01–0.015)	0.009 ± 0.005<br>(0.008–0.011)	99.7 ± 72<br>(79.3–120.2)	83.8 ± 75.5<br>(61.7–106)	73.8 ± 45.3<br>(60.9–86.7)
**Cheek**	**T0**	0.02 ± 0.008<br>(0.017–0.022)	0.021 ± 0.008<br>(0.018–0.023)	0.019 ± 0.01<br>(0.016–0.022)	100 ± 0<br>(100–100)	100 ± 0<br>(100–100)	100 ± 0<br>(100–100)
	**M1**	0.02 ± 0.01<br>(0.018–0.023)	0.019 ± 0.008<br>(0.016–0.021)	0.019 ± 0.009<br>(0.017–0.022)	113.8 ± 56.4<br>(97.8–129.8)	99.9 ± 47.9<br>(86.1–113.6)	120.2 ± 68.4<br>(100.8–139.7)
	**M2**	0.018 ± 0.007<br>(0.016–0.02)	0.02 ± 0.009<br>(0.017–0.022)	0.017 ± 0.007<br>(0.015–0.019)	101.1 ± 43.8<br>(88.7–113.6)	109.2 ± 53.2<br>(93.7–124.6)	105.4 ± 50<br>(91.1–119.6)
	**M3**	0.013 ± 0.01<br>(0.011–0.016)	0.013 ± 0.005<br>(0.011–0.014)	0.012 ± 0.007<br>(0.01–0.014)	80.1 ± 70.1<br>(60.2–100)	73.4 ± 48<br>(59.3–87.5)	74.1 ± 47<br>(60.8–87.5)
**ELASTICITY – R2**							
**Site**	**Time point**	**Ratio/unitless**			**%T0<br>mean ± SD (95% CI)**		
		**Placebo**	**SH60**	**SH120**	**Placebo**	**SH60**	**SH120**

**Forehead**	**T0**	0.526 ± 0.074 (0.505–0.547)	0.521 ± 0.076 (0.499–0.543)	0.55 ± 0.07 (0.53–0.57)	100 ± 0 (100–100)	100 ± 0 (100–100)	100 ± 0 (100–100)
	**M1**	0.462 ± 0.092 (0.436–0.488)	0.482 ± 0.095 (0.454–0.509)	0.454 ± 0.094 (0.427–0.481)	89.3 ± 20.1 (83.6–95)	93.9 ± 21.8 (87.6–100.1)	83.7 ± 20 (78–89.4)
	**M2**	0.377 ± 0.078 (0.355–0.399)	0.379 ± 0.074 (0.358–0.401)	0.39 ± 0.078 (0.368–0.413)	73 ± 17.5 (68–78)	74.8 ± 19.3 (69.1–80.4)	71.5 ± 14.1 (67.5–75.5)
	**M3**	0.388 ± 0.091 (0.362–0.414)	0.376 ± 0.082 (0.352–0.4)	0.412 ± 0.081 (0.389–0.435)	74.8 ± 17.4 (69.9–79.7)	73.6 ± 17.7 (68.4–78.8)	74.8 ± 13.5 (71–78.6)
**Cheek**	**T0**	0.57 ± 0.072 (0.549–0.59)	0.577 ± 0.075 (0.556–0.599)	0.587 ± 0.08 (0.565–0.61)	100 ± 0 (100–100)	100 ± 0 (100–100)	100 ± 0 (100–100)
	**M1**	0.465 ± 0.106 (0.435–0.495)	0.468 ± 0.088 (0.443–0.493)	0.474 ± 0.099 (0.446–0.502)	82.2 ± 19 (76.8–87.6)	81.8 ± 15.8 (77.2–86.3)	82.8 ± 24 (76–89.7)
	**M2**	0.401 ± 0.071 (0.381–0.421)	0.382 ± 0.082 (0.359–0.406)	0.402 ± 0.076 (0.38–0.424)	71.2 ± 13.8 (67.3–75.1)	67.2 ± 15.8 (62.6–71.8)	69.2 ± 14 (65.2–73.2)
	**M3**	0.409 ± 0.083 (0.385–0.433)	0.382 ± 0.067 (0.362–0.401)	0.422 ± 0.083 (0.398–0.446)	72.2 ± 13.5 (68.4–76.1)	67.4 ± 13.8 (63.3–71.4)	72.4 ± 13.4 (68.6–76.2)
**ELASTICITY – R3**							
**Site**	**Time point**	**mm**			**%T0 mean ± SD (95% CI)**		
		**Placebo**	**SH60**	**SH120**	**Placebo**	**SH60**	**SH120**
**Forehead**	**T0**	0.042 ± 0.019 (0.037–0.048)	0.047 ± 0.024 (0.04–0.054)	0.043 ± 0.021 (0.037–0.049)	100 ± 0 (100–100)	100 ± 0 (100–100)	100 ± 0 (100–100)
	**M1**	0.036 ± 0.019 (0.03–0.041)	0.036 ± 0.019 (0.03–0.041)	0.036 ± 0.02 (0.03–0.042)	87.9 ± 37.2 (77.3–98.4)	81.5 ± 37.4 (70.8–92.3)	86.6 ± 32.8 (77.3–95.9)
	**M2**	0.031 ± 0.015 (0.027–0.035)	0.027 ± 0.011 (0.024–0.03)	0.029 ± 0.016 (0.024–0.033)	81 ± 37.4 (70.4–91.6)	67.8 ± 28.8 (59.5–76.2)	73.2 ± 34.1 (63.5–82.9)
	**M3**	0.023 ± 0.012 (0.019–0.026)	0.021 ± 0.011 (0.017–0.024)	0.017 ± 0.007 (0.015–0.019)	61.5 ± 36.2 (51.2–71.8)	51.5 ± 35.2 (41.2–61.9)	46.2 ± 23.1 (39.7–52.8)
**Cheek**	**T0**	0.057 ± 0.02 (0.051–0.063)	0.06 ± 0.021 (0.054–0.067)	0.059 ± 0.03 (0.05–0.067)	100 ± 0 (100–100)	100 ± 0 (100–100)	100 ± 0 (100–100)
	**M1**	0.046 ± 0.023 (0.04–0.053)	0.043 ± 0.022 (0.037–0.049)	0.045 ± 0.025 (0.038–0.052)	82.2 ± 29.2 (73.9–90.5)	72.3 ± 24.2 (65.3–79.2)	80.5 ± 29.6 (72.1–88.9)
	**M2**	0.033 ± 0.01 (0.03–0.036)	0.034 ± 0.011 (0.031–0.038)	0.032 ± 0.011 (0.029–0.035)	62.9 ± 25.1 (55.7–70)	61.8 ± 21.1 (55.6–67.9)	62.1 ± 26.3 (54.7–69.6)
	**M3**	0.024 ± 0.015 (0.02–0.028)	0.023 ± 0.008 (0.02–0.025)	0.023 ± 0.011 (0.02–0.026)	49.1 ± 38.5 (38.2–60.1)	42.4 ± 20.9 (36.2–48.5)	45.8 ± 24.6 (38.8–52.7)
**ELASTICITY – R4**							
**Site**	**Time point**	**mm**			**%T0 mean ± SD (95% CI)**		
		**Placebo**	**SH60**	**SH120**	**Placebo**	**SH60**	**SH120**
**Forehead**	**T0**	0.024 ± 0.011 (0.021–0.027)	0.027 ± 0.014 (0.023–0.032)	0.024 ± 0.011 (0.02–0.027)	100 ± 0 (100–100)	100 ± 0 (100–100)	100 ± 0 (100–100)
	**M1**	0.023 ± 0.011 (0.02–0.026)	0.023 ± 0.011 (0.02–0.026)	0.022 ± 0.011 (0.019–0.025)	104.6 ± 60.3 (87.4–121.7)	100 ± 79.3 (77.3–122.8)	102.3 ± 44.8 (89.6–115.1)
	**M2**	0.024 ± 0.013 (0.02–0.028)	0.021 ± 0.009 (0.018–0.024)	0.022 ± 0.014 (0.018–0.026)	110.1 ± 55.5 (94.3–125.8)	91.9 ± 45.8 (78.7–105.2)	101.1 ± 53.4 (85.9–116.3)
	**M3**	0.017 ± 0.01 (0.015–0.02)	0.016 ± 0.01 (0.013–0.019)	0.013 ± 0.006 (0.011–0.014)	83.3 ± 53.1 (68.3–98.4)	70.9 ± 51.6 (55.7–86)	62.1 ± 33.7 (52.5–71.7)
**Cheek**	**T0**	0.03 ± 0.011 (0.027–0.033)	0.032 ± 0.012 (0.028–0.035)	0.029 ± 0.014 (0.025–0.033)	100 ± 0 (100–100)	100 ± 0 (100–100)	100 ± 0 (100–100)
	**M1**	0.029 ± 0.012 (0.025–0.032)	0.026 ± 0.011 (0.023–0.029)	0.027 ± 0.013 (0.024–0.031)	104.1 ± 48.5 (90.4–117.9)	89.8 ± 38.2 (78.9–100.8)	105.8 ± 51.6 (91.1–120.5)
	**M2**	0.024 ± 0.009 (0.022–0.027)	0.026 ± 0.01 (0.023–0.029)	0.023 ± 0.009 (0.021–0.026)	89.1 ± 38.5 (78.2–100.1)	91.8 ± 41.1 (79.8–103.7)	91.3 ± 41.1 (79.6–102.9)
	**M3**	0.018 ± 0.013 (0.014–0.021)	0.017 ± 0.007 (0.015–0.019)	0.016 ± 0.009 (0.014–0.019)	69.8 ± 62.3 (52.1–87.5)	63.6 ± 40.1 (51.8–75.3)	66 ± 40.7 (54.5–77.6)
**ELASTICITY – R5**							
**Site**	**Time point**	**Ratio/unitless**			**%T0 mean ± SD (95% CI)**		
		**Placebo**	**SH60**	**SH120**	**Placebo**	**SH60**	**SH120**
**Forehead**	**T0**	0.433 ± 0.127 (0.397–0.469)	0.448 ± 0.144 (0.406–0.489)	0.456 ± 0.121 (0.422–0.491)	100 ± 0 (100–100)	100 ± 0 (100–100)	100 ± 0 (100–100)
	**M1**	0.33 ± 0.136 (0.291–0.368)	0.329 ± 0.13 (0.291–0.366)	0.315 ± 0.13 (0.278–0.352)	83.8 ± 47.2 (70.3–97.4)	80.6 ± 43.5 (68.1–93)	72.8 ± 31.7 (63.8–81.8)
	**M2**	0.204 ± 0.07 (0.184–0.224)	0.21 ± 0.084 (0.186–0.234)	0.222 ± 0.086 (0.197–0.246)	48.8 ± 20.2 (43–54.6)	52.1 ± 31.7 (42.9–61.3)	50.3 ± 18.3 (45.1–55.5)
	**M3**	0.201 ± 0.082 (0.177–0.224)	0.222 ± 0.102 (0.193–0.252)	0.252 ± 0.1 (0.224–0.28)	47.6 ± 19 (42.1–53)	53 ± 24.7 (45.7–60.2)	57.7 ± 26.7 (50.1–65.3)
**Cheek**	**T0**	0.444 ± 0.115 (0.411–0.476)	0.44 ± 0.129 (0.403–0.477)	0.469 ± 0.134 (0.431–0.507)	100 ± 0 (100–100)	100 ± 0 (100–100)	100 ± 0 (100–100)
	**M1**	0.292 ± 0.141 (0.252–0.332)	0.288 ± 0.128 (0.251–0.324)	0.306 ± 0.139 (0.266–0.345)	68.2 ± 32.1 (59–77.3)	68.2 ± 31.4 (59.2–77.3)	72.3 ± 45.8 (59.3–85.3)
	**M2**	0.181 ± 0.062 (0.164–0.199)	0.178 ± 0.066 (0.159–0.197)	0.197 ± 0.096 (0.17–0.225)	42.6 ± 16.1 (38.1–47.2)	43.7 ± 19.6 (38–49.4)	43.8 ± 19.7 (38.2–49.4)
	**M3**	0.217 ± 0.076 (0.196–0.239)	0.195 ± 0.066 (0.176–0.214)	0.231 ± 0.104 (0.201–0.26)	50.8 ± 17.5 (45.8–55.8)	48.1 ± 21.2 (41.9–54.4)	51.8 ± 21.8 (45.6–58)
**ELASTICITY – R6**							

Site	Time point	Ratio/unitless			%T0 mean ± SD (95% CI)		
		Placebo	SH60	SH120	Placebo	SH60	SH120
Forehead	T0	0.812 ± 0.227 (0.747–0.876)	0.802 ± 0.238 (0.734–0.871)	0.81 ± 0.266 (0.735–0.886)	100 ± 0 (100–100)	100 ± 0 (100–100)	100 ± 0 (100–100)
	M1	0.575 ± 0.255 (0.502–0.647)	0.603 ± 0.263 (0.527–0.678)	0.545 ± 0.205 (0.486–0.603)	79.4 ± 48.1 (65.7–93.1)	81.6 ± 44.5 (68.8–94.4)	73.8 ± 32.3 (64.6–82.9)
	M2	0.388 ± 0.13 (0.351–0.425)	0.391 ± 0.141 (0.35–0.432)	0.406 ± 0.185 (0.353–0.458)	50.4 ± 20.8 (44.5–56.3)	53.4 ± 29.9 (44.7–62.1)	52.2 ± 19.6 (46.6–57.8)
	M3	0.36 ± 0.161 (0.314–0.405)	0.406 ± 0.163 (0.359–0.453)	0.436 ± 0.158 (0.391–0.481)	46 ± 18.5 (40.7–51.3)	52.9 ± 20.6 (46.8–59)	57.5 ± 25 (50.4–64.7)
Cheek	T0	0.678 ± 0.221 (0.615–0.741)	0.656 ± 0.18 (0.604–0.708)	0.67 ± 0.17 (0.622–0.719)	100 ± 0 (100–100)	100 ± 0 (100–100)	100 ± 0 (100–100)
	M1	0.459 ± 0.209 (0.4–0.518)	0.464 ± 0.206 (0.405–0.524)	0.494 ± 0.247 (0.424–0.564)	71 ± 31.4 (62–79.9)	72.8 ± 30.7 (63.9–81.6)	78.3 ± 44 (65.8–90.8)
	M2	0.273 ± 0.106 (0.243–0.303)	0.279 ± 0.096 (0.251–0.307)	0.301 ± 0.149 (0.259–0.343)	42.1 ± 15.7 (37.7–46.6)	45.7 ± 20.9 (39.7–51.8)	45.7 ± 18.3 (40.5–50.9)
	M3	0.313 ± 0.101 (0.284–0.341)	0.31 ± 0.11 (0.278–0.342)	0.348 ± 0.127 (0.311–0.384)	48.3 ± 14.7 (44.1–52.5)	49.8 ± 20.4 (43.8–55.8)	54.4 ± 21.9 (48.2–60.6)

ELASTICITY – R7

Site	Time point	Ratio/unitless			%T0 mean ± SD (95% CI)		
		Placebo	SH60	SH120	Placebo	SH60	SH120
Forehead	T0	0.235 ± 0.053 (0.22–0.25)	0.242 ± 0.053 (0.226–0.257)	0.247 ± 0.042 (0.235–0.259)	100 ± 0 (100–100)	100 ± 0 (100–100)	100 ± 0 (100–100)
	M1	0.2 ± 0.059 (0.183–0.217)	0.197 ± 0.054 (0.181–0.212)	0.194 ± 0.065 (0.175–0.212)	88.6 ± 34.9 (78.6–98.7)	84.8 ± 29.7 (76.3–93.3)	79.8 ± 27.1 (72.1–87.4)
	M2	0.141 ± 0.039 (0.13–0.152)	0.146 ± 0.045 (0.133–0.159)	0.152 ± 0.045 (0.139–0.165)	61 ± 19.9 (55.3–66.7)	63.2 ± 25.9 (55.7–70.7)	62.3 ± 18.9 (57–67.7)
	M3	0.14 ± 0.044 (0.128–0.153)	0.15 ± 0.055 (0.134–0.166)	0.168 ± 0.052 (0.153–0.183)	59.8 ± 17.5 (54.8–64.8)	64 ± 24.2 (56.9–71.1)	68.9 ± 23.9 (62.1–75.7)
Cheek	T0	0.261 ± 0.049 (0.247–0.275)	0.261 ± 0.056 (0.245–0.277)	0.275 ± 0.059 (0.259–0.292)	100 ± 0 (100–100)	100 ± 0 (100–100)	100 ± 0 (100–100)
	M1	0.191 ± 0.066 (0.172–0.209)	0.189 ± 0.064 (0.171–0.207)	0.196 ± 0.06 (0.179–0.213)	74.7 ± 26.8 (67–82.3)	74.5 ± 28.8 (66.3–82.8)	75.2 ± 33.1 (65.8–84.6)
	M2	0.138 ± 0.041 (0.126–0.149)	0.136 ± 0.041 (0.124–0.147)	0.147 ± 0.048 (0.133–0.16)	53.8 ± 16.4 (49.2–58.5)	54 ± 18.5 (48.6–59.3)	54.5 ± 17.4 (49.5–59.4)
	M3	0.161 ± 0.047 (0.148–0.175)	0.146 ± 0.04 (0.135–0.158)	0.165 ± 0.053 (0.15–0.18)	62.8 ± 17.8 (57.8–67.9)	58.7 ± 20.5 (52.7–64.7)	61.2 ± 18.4 (55.9–66.4)

ELASTICITY – R9

Site	Time point	mm			%T0 mean ± SD (95% CI)		
		Placebo	SH60	SH120	Placebo	SH60	SH120
Forehead	T0	0.009 ± 0.003 (0.008–0.01)	0.01 ± 0.004 (0.008–0.011)	0.009 ± 0.004 (0.008–0.011)	100 ± 0 (100–100)	100 ± 0 (100–100)	100 ± 0 (100–100)
	M1	0.006 ± 0.005 (0.004–0.007)	0.006 ± 0.004 (0.004–0.007)	0.006 ± 0.005 (0.005–0.007)	60.2 ± 36.2 (49.9–70.5)	58.4 ± 33.3 (48.9–68)	62.5 ± 32.2 (53.3–71.6)
	M2	0.003 ± 0.001 (0.003–0.003)	0.003 ± 0.001 (0.003–0.003)	0.003 ± 0.001 (0.003–0.003)	37 ± 16 (32.5–41.5)	33.6 ± 14.9 (29.3–38)	38.5 ± 20.8 (32.6–44.4)
	M3	0.002 ± 0.001 (0.002–0.002)	0.002 ± 0.001 (0.002–0.002)	0.002 ± 0.001 (0.002–0.002)	24.7 ± 12.5 (21.2–28.3)	26.1 ± 14.6 (21.8–30.4)	22.6 ± 10.6 (19.6–25.6)
Cheek	T0	0.011 ± 0.004 (0.01–0.012)	0.012 ± 0.005 (0.01–0.013)	0.012 ± 0.009 (0.01–0.015)	100 ± 0 (100–100)	100 ± 0 (100–100)	100 ± 0 (100–100)
	M1	0.007 ± 0.005 (0.005–0.008)	0.007 ± 0.005 (0.005–0.008)	0.007 ± 0.006 (0.006–0.009)	58.5 ± 31.9 (49.5–67.6)	59.3 ± 33.3 (49.8–68.9)	62.7 ± 37.4 (52.1–73.3)
	M2	0.003 ± 0.001 (0.003–0.003)	0.003 ± 0.001 (0.003–0.003)	0.004 ± 0.004 (0.003–0.005)	28.6 ± 12.5 (25–32.1)	26.9 ± 12 (23.5–30.4)	31 ± 14.4 (26.9–35.1)
	M3	0.002 ± 0.001 (0.002–0.003)	0.003 ± 0.001 (0.002–0.003)	0.003 ± 0.003 (0.002–0.004)	22.8 ± 11.9 (19.4–26.2)	25.1 ± 13.9 (21–29.1)	25.3 ± 12.5 (21.7–28.8)

REDNESS AREAS

Site	Time point	Area (pix^2)			%T0 mean ± SD (95% CI)		
		Placebo	SH60	SH120	Placebo	SH60	SH120
Forehead	T0	83.1 ± 236 (16.1–150.2)	60.3 ± 63 (42.1–78.4)	77.9 ± 138.6 (38.5–117.3)	100 ± 0 (100–100)	100 ± 0 (100–100)	100 ± 0 (100–100)
	M1	64.2 ± 94.9 (37.3–91.2)	46.5 ± 44.4 (33.7–59.2)	46.7 ± 53.3 (31.4–62)	122.2 ± 118 (88.7–155.7)	93 ± 43.8 (80.4–105.5)	102.2 ± 78.3 (79.7–124.7)
	M2	66.6 ± 91.6 (40.6–92.6)	67.3 ± 105.6 (36.6–97.9)	61.8 ± 83.8 (38–85.6)	129.6 ± 133.6 (91.6–167.6)	123.6 ± 103.4 (93.5–153.6)	120.8 ± 93.2 (94.3–147.3)
	M3	64.4 ± 127.3 (28.2–100.5)	65.3 ± 112 (32.5–98.2)	53.7 ± 69.3 (34–73.4)	149 ± 267.7 (72.9–225.1)	124.7 ± 110.3 (92.6–156.7)	112.1 ± 86.3 (87.6–136.6)
Cheek	T0	893.2 ± 4320.4 (-334.7–2121)	295.3 ± 312.4 (205.6–385)	842.3 ± 3095.1 (-37.3–1721.9)	100 ± 0 (100–100)	100 ± 0 (100–100)	100 ± 0 (100–100)
	M1	254.8 ± 280.5 (175.1–334.5)	319.6 ± 429 (196.4–442.8)	321.4 ± 493.6 (178.1–464.7)	130.1 ± 210.2 (70.4–189.8)	129.9 ± 90.6 (103.9–155.9)	102.8 ± 69.1 (82.8–122.9)
	M2	309.4 ± 482.7 (172.3–446.6)	425.1 ± 1008.5 (132.2–717.9)	293 ± 322.6 (200.3–385.6)	132.6 ± 160.3 (87–178.2)	119.5 ± 86.5 (94.4–144.6)	121.6 ± 99.9 (92.9–150.3)

	M3	346.9 ± 620.4<br>(170.6–523.2)	411.2 ± 692.3<br>(207.9–614.5)	308.7 ± 405.4<br>(192.3–425.2)	160.8 ± 245.2<br>(91.1–230.4)	140.4 ± 136.7<br>(100.7–180.1)	109.8 ± 90.4<br>(83.9–135.8)
**PORE SIZE**							
**Site**	**Time point**	**Area (pix^2)**			**%T0<br>mean ± SD (95% CI)**		
		**Placebo**	**SH60**	**SH120**	**Placebo**	**SH60**	**SH120**
**Forehead**	**T0**	5.4 ± 1.6<br>(5–5.9)	19.6 ± 102.4<br>(-9.8–49)	27.7 ± 159.8<br>(-17.7–73.1)	100 ± 0<br>(100–100)	100 ± 0<br>(100–100)	100 ± 0<br>(100–100)
	**M1**	5.1 ± 1.3<br>(4.8–5.5)	37.6 ± 229.6<br>(-28.3–103.6)	31 ± 183.1<br>(-21.1–83)	97 ± 15.7<br>(92.5–101.4)	101.8 ± 26.6<br>(94.1–109.4)	98.6 ± 19.7<br>(93–104.3)
	**M2**	5.2 ± 1.3<br>(4.8–5.6)	25 ± 139.6<br>(-15.6–65.5)	30.5 ± 181.5<br>(-21.1–82)	99.6 ± 24.7<br>(92.6–106.6)	100.7 ± 27.9<br>(92.6–108.8)	97.6 ± 22.6<br>(91.2–104)
	**M3**	5.3 ± 1.8<br>(4.8–5.8)	19.5 ± 98.2<br>(-9.4–48.3)	24 ± 136.4<br>(-14.8–62.7)	99.8 ± 27.2<br>(92–107.5)	104.6 ± 50.3<br>(89.8–119.4)	94.2 ± 18.4<br>(89–99.4)
**Cheek**	**T0**	7.4 ± 3.3<br>(6.5–8.3)	8.1 ± 4<br>(7–9.3)	7 ± 2.1<br>(6.4–7.6)	100 ± 0<br>(100–100)	100 ± 0<br>(100–100)	100 ± 0<br>(100–100)
	**M1**	6.9 ± 2.4<br>(6.2–7.6)	7.6 ± 3.3<br>(6.6–8.5)	7.4 ± 2.5<br>(6.7–8.1)	96.6 ± 17.9<br>(91.5–101.7)	97.7 ± 19.4<br>(92.1–103.2)	106.3 ± 19.3<br>(100.8–111.8)
	**M2**	7.3 ± 3.2<br>(6.4–8.2)	7.3 ± 2.7<br>(6.6–8.1)	7.2 ± 2.7<br>(6.4–8)	99.5 ± 19.4<br>(94–105)	98.9 ± 27.3<br>(91–106.8)	103.4 ± 21.2<br>(97.3–109.4)
	**M3**	7.6 ± 3.7<br>(6.5–8.6)	7.4 ± 3.1<br>(6.5–8.3)	7.3 ± 2.7<br>(6.5–8)	102.2 ± 21.5<br>(96.1–108.3)	98.1 ± 25.9<br>(90.5–105.7)	104.1 ± 22.7<br>(97.7–110.6)
**ERYTHEMA INDEX**							
**Site**	**Time point**	**A.U.**			**%T0<br>mean ± SD (95% CI)**		
		**Placebo**	**SH60**	**SH120**	**Placebo**	**SH60**	**SH120**
**Forehead**	**T0**	417.1 ± 76.5<br>(395.3–438.8)	454 ± 74.3<br>(432.6–475.3)	435.9 ± 67<br>(416.8–454.9)	100 ± 0<br>(100–100)	100 ± 0<br>(100–100)	100 ± 0<br>(100–100)
	**M1**	394.7 ± 74.6<br>(373.5–416)	423.6 ± 66<br>(404.7–442.6)	406.2 ± 64.8<br>(387.8–424.6)	94.8 ± 7<br>(92.8–96.8)	93.6 ± 6.8<br>(91.7–95.6)	93.8 ± 11.9<br>(90.4–97.2)
	**M2**	380.8 ± 78.3<br>(358.6–403.1)	409.5 ± 72.6<br>(388.4–430.6)	399.4 ± 66.2<br>(380.6–418.2)	91.4 ± 10<br>(88.5–94.2)	90.4 ± 8.1<br>(88.1–92.8)	91.9 ± 8.5<br>(89.5–94.3)
	**M3**	360.5 ± 73<br>(339.8–381.3)	394.4 ± 71.5<br>(373.4–415.4)	381 ± 64.2<br>(362.7–399.2)	86.7 ± 10.9<br>(83.6–89.8)	87.1 ± 6.5<br>(85.2–89)	87.6 ± 7.9<br>(85.3–89.8)
**Cheek**	**T0**	450.1 ± 69.4<br>(430.3–469.8)	471.2 ± 81.9<br>(447.7–494.8)	472.2 ± 72.7<br>(451.6–492.9)	100 ± 0<br>(100–100)	100 ± 0<br>(100–100)	100 ± 0<br>(100–100)
	**M1**	424 ± 72.4<br>(403.5–444.6)	447.1 ± 74.4<br>(425.7–468.4)	448.5 ± 75.8<br>(427–470.1)	94.3 ± 8<br>(92–96.6)	95.2 ± 6.8<br>(93.2–97.2)	95.1 ± 8.2<br>(92.7–97.4)
	**M2**	402.4 ± 70.8<br>(382.3–422.5)	431 ± 72.2<br>(410–452)	426.6 ± 75.3<br>(405.2–448)	89.7 ± 11<br>(86.6–92.8)	91.5 ± 6.7<br>(89.5–93.4)	90.5 ± 9<br>(87.9–93)
	**M3**	392 ± 73.4<br>(371.1–412.8)	416.6 ± 78.1<br>(393.7–439.5)	409.4 ± 78.7<br>(387–431.7)	87.1 ± 9.1<br>(84.5–89.7)	88.2 ± 7.9<br>(85.9–90.5)	86.5 ± 8.5<br>(84.1–88.9)
**MELANIN INDEX**							
**Site**	**Time point**	**A.U.**			**%T0<br>mean ± SD (95% CI)**		
		**Placebo**	**SH60**	**SH120**	**Placebo**	**SH60**	**SH120**
**Forehead**	**T0**	140.3 ± 34.2<br>(130.6–150)	143.5 ± 29<br>(135.1–151.8)	142.8 ± 28.1<br>(134.8–150.8)	100 ± 0<br>(100–100)	100 ± 0<br>(100–100)	100 ± 0<br>(100–100)
	**M1**	135.9 ± 27.5<br>(128.1–143.7)	144.2 ± 27.1<br>(136.4–152)	142.4 ± 27.2<br>(134.6–150.1)	98.3 ± 10<br>(95.4–101.1)	101.1 ± 8.6<br>(98.7–103.6)	100.2 ± 8.6<br>(97.8–102.7)
	**M2**	133.3 ± 21.1<br>(127.3–139.2)	140.4 ± 24.5<br>(133.3–147.5)	139.6 ± 23.5<br>(132.9–146.2)	97.5 ± 13.6<br>(93.6–101.4)	99.1 ± 8.7<br>(96.6–101.7)	98.7 ± 10.2<br>(95.8–101.6)
	**M3**	132 ± 22.6<br>(125.6–138.4)	138.9 ± 23.8<br>(131.9–145.9)	137.1 ± 21.1<br>(131.1–143)	96.3 ± 13<br>(92.6–100)	97.9 ± 9.6<br>(95.1–100.7)	97.4 ± 11.6<br>(94.1–100.7)
**Cheek**	**T0**	118.1 ± 32.5<br>(108.8–127.3)	121.4 ± 28.6<br>(113.2–129.6)	128.3 ± 37.1<br>(117.8–138.9)	100 ± 0<br>(100–100)	100 ± 0<br>(100–100)	100 ± 0<br>(100–100)
	**M1**	112.9 ± 24.8<br>(105.8–119.9)	120.9 ± 27.5<br>(113–128.8)	124.1 ± 31.9<br>(115.1–133.2)	97.5 ± 13<br>(93.8–101.2)	100.5 ± 13.8<br>(96.5–104.4)	98.7 ± 14.6<br>(94.5–102.9)
	**M2**	111.1 ± 24.9<br>(104–118.2)	117.6 ± 23.9<br>(110.6–124.5)	122.4 ± 28.4<br>(114.3–130.5)	96.1 ± 14.1<br>(92.1–100.1)	99.7 ± 17.1<br>(94.8–104.7)	98.5 ± 21.1<br>(92.5–104.5)
	**M3**	107.9 ± 24.5<br>(100.9–114.9)	114.2 ± 22.5<br>(107.6–120.8)	116.6 ± 25.5<br>(109.4–123.9)	93.4 ± 15.2<br>(89.1–97.7)	96.9 ± 17.7<br>(91.7–102.1)	93.8 ± 15.6<br>(89.3–98.2)
**ITA°**							
**Site**	**Time point**	**°**			**%T0<br>mean ± SD (95% CI)**		
		**Placebo**	**SH60**	**SH120**	**Placebo**	**SH60**	**SH120**
**Forehead**	**T0**	42.9 ± 9.5<br>(40.2–45.5)	39.9 ± 8.6<br>(37.5–42.4)	40.5 ± 8.9<br>(37.9–43)	100 ± 0<br>(100–100)	100 ± 0<br>(100–100)	100 ± 0<br>(100–100)
	**M1**	45.5 ± 8.3<br>(43.1–47.9)	42.4 ± 7.3<br>(40.4–44.5)	43.3 ± 7.7<br>(41.1–45.4)	107.7 ± 10.6<br>(104.7–110.7)	109.4 ± 21.4<br>(103.3–115.5)	109 ± 15.2<br>(104.7–113.3)
	**M2**	47.2 ± 7.3<br>(45.1–49.3)	44.7 ± 7.2<br>(42.6–46.8)	44.8 ± 7.4<br>(42.7–46.9)	112.6 ± 14.7<br>(108.4–116.7)	116 ± 27.8<br>(107.9–124.1)	112.9 ± 12.8<br>(109.3–116.6)
	**M3**	49.2 ± 6.9<br>(47.2–51.1)	46.4 ± 6.7<br>(44.4–48.3)	46.5 ± 6.6<br>(44.6–48.3)	117.8 ± 17.6<br>(112.8–122.9)	121.3 ± 32.8<br>(111.7–130.9)	118.4 ± 20.1<br>(112.7–124.1)
**Cheek**	**T0**	30.5 ± 11.2<br>(27.3–33.6)	29.5 ± 12.7<br>(25.9–33.2)	27.7 ± 11.7<br>(24.4–31)	100 ± 0<br>(100–100)	100 ± 0<br>(100–100)	100 ± 0<br>(100–100)

	**M1**	34.4 ± 10 (31.6–37.3)	32.3 ± 11.1 (29.1–35.5)	30.5 ± 11.8 (27.2–33.9)	121.2 ± 43 (108.8–133.5)	115.9 ± 35 (105.6–126.1)	111.2 ± 23.9 (104.4–117.9)
	**M2**	36.6 ± 9.9 (33.8–39.4)	35.2 ± 10.2 (32.2–38.2)	33.1 ± 11.5 (29.9–36.4)	128.6 ± 42.8 (116.3–140.9)	128.2 ± 49.1 (113.6–142.8)	122 ± 33.2 (112.6–131.4)
	**M3**	39 ± 8.8 (36.5–41.5)	37.8 ± 10.2 (34.8–40.8)	36.6 ± 10.3 (33.6–39.5)	143 ± 68.8 (123.2–162.7)	139.7 ± 52.6 (123.9–155.5)	138.2 ± 66.1 (119.4–157)
**a* VALUE**							
**Site**	**Time point**	**Unitless**			**%T0 mean ± SD (95% CI)**		
		**Placebo**	**SH60**	**SH120**	**Placebo**	**SH60**	**SH120**
**Forehead**	**T0**	13.2 ± 1.6 (12.8–13.7)	13.9 ± 1.8 (13.3–14.4)	13.5 ± 1.6 (13–13.9)	100 ± 0 (100–100)	100 ± 0 (100–100)	100 ± 0 (100–100)
	**M1**	12.7 ± 1.6 (12.3–13.2)	13.4 ± 1.8 (12.9–13.9)	12.9 ± 1.7 (12.5–13.4)	96.4 ± 5.9 (94.7–98.1)	96.6 ± 4.7 (95.2–97.9)	96.1 ± 7.5 (94–98.2)
	**M2**	12.5 ± 1.7 (12–12.9)	13.1 ± 1.8 (12.6–13.6)	12.8 ± 1.6 (12.3–13.3)	94.3 ± 6.8 (92.3–96.2)	94.5 ± 6.1 (92.7–96.3)	94.9 ± 6.1 (93.1–96.6)
	**M3**	12.2 ± 1.7 (11.7–12.7)	12.9 ± 1.9 (12.3–13.5)	12.5 ± 1.7 (12–13)	92 ± 6.7 (90.1–93.9)	93.1 ± 8.3 (90.7–95.6)	92.4 ± 5.7 (90.8–94)
**Cheek**	**T0**	16.3 ± 1.6 (15.8–16.7)	16.6 ± 1.8 (16.1–17.1)	16.5 ± 1.6 (16–17)	100 ± 0 (100–100)	100 ± 0 (100–100)	100 ± 0 (100–100)
	**M1**	16 ± 1.5 (15.6–16.4)	16.4 ± 2 (15.8–16.9)	16.2 ± 1.7 (15.7–16.7)	98.7 ± 5.4 (97.1–100.2)	98.7 ± 6.4 (96.8–100.5)	98.2 ± 5.9 (96.5–99.8)
	**M2**	15.7 ± 1.7 (15.3–16.2)	16.1 ± 1.8 (15.6–16.6)	16 ± 1.8 (15.5–16.6)	96.9 ± 6.4 (95.1–98.7)	96.6 ± 6 (94.9–98.4)	97.2 ± 6.2 (95.4–99)
	**M3**	15.3 ± 1.7 (14.8–15.8)	15.7 ± 1.9 (15.2–16.3)	15.5 ± 1.7 (15–16)	94.2 ± 6.7 (92.3–96.1)	94.5 ± 6.7 (92.6–96.5)	93.8 ± 6.4 (92–95.6)
**b* VALUE**							
**Site**	**Time point**	**Unitless**			**%T0 mean ± SD (95% CI)**		
		**Placebo**	**SH60**	**SH120**	**Placebo**	**SH60**	**SH120**
**Forehead**	**T0**	11.7 ± 1.7 (11.2–12.1)	12.1 ± 1.7 (11.6–12.6)	12 ± 1.6 (11.5–12.5)	100 ± 0 (100–100)	100 ± 0 (100–100)	100 ± 0 (100–100)
	**M1**	11.6 ± 1.6 (11.1–12)	12.1 ± 1.7 (11.6–12.6)	12.1 ± 1.7 (11.6–12.6)	99.7 ± 7.1 (97.7–101.7)	100.6 ± 5.9 (98.9–102.2)	100.9 ± 6.7 (99–102.8)
	**M2**	11.4 ± 1.5 (11–11.8)	11.7 ± 1.6 (11.2–12.2)	11.9 ± 1.6 (11.4–12.3)	98.2 ± 8.3 (95.8–100.5)	97.4 ± 7.6 (95.2–99.6)	99.1 ± 7.8 (96.9–101.3)
	**M3**	11.2 ± 1.4 (10.8–11.6)	11.7 ± 1.6 (11.2–12.1)	11.8 ± 1.6 (11.4–12.3)	96.7 ± 8.4 (94.4–99.1)	97.4 ± 7.8 (95.1–99.7)	99.2 ± 8.8 (96.7–101.7)
**Cheek**	**T0**	11.6 ± 1.5 (11.2–12)	11.5 ± 1.6 (11.1–12)	12 ± 1.9 (11.5–12.6)	100 ± 0 (100–100)	100 ± 0 (100–100)	100 ± 0 (100–100)
	**M1**	11.4 ± 1.6 (11–11.8)	11.5 ± 1.6 (11–12)	11.7 ± 1.9 (11.1–12.2)	98.4 ± 5.3 (96.9–99.9)	99.9 ± 6.7 (98–101.8)	97 ± 6.7 (95.1–99)
	**M2**	11.1 ± 1.4 (10.7–11.5)	11.1 ± 1.5 (10.7–11.6)	11.5 ± 1.8 (10.9–12)	96.1 ± 7 (94.1–98)	97.3 ± 8.1 (95–99.7)	95.6 ± 7.9 (93.3–97.8)
	**M3**	11 ± 1.5 (10.6–11.5)	11 ± 1.6 (10.5–11.5)	11.5 ± 1.8 (11–12)	95.6 ± 9.1 (93–98.1)	96.4 ± 8.3 (94–98.9)	96 ± 8.3 (93.6–98.4)
**GLOSS (DSC)**							
**Site**	**Time point**	**G.U. (gloss units)**			**%T0 mean ± SD (95% CI)**		
		**Placebo**	**SH60**	**SH120**	**Placebo**	**SH60**	**SH120**
**Forehead**	**T0**	6 ± 2.2 (5.3–6.6)	5.7 ± 1.9 (5.2–6.2)	5.6 ± 1.9 (5–6.1)	100 ± 0 (100–100)	100 ± 0 (100–100)	100 ± 0 (100–100)
	**M1**	5.5 ± 1.7 (5.0–6.0)	5.6 ± 1.9 (5–6.1)	5.4 ± 1.8 (4.9–5.9)	99.1 ± 36 (88.9–109.4)	102 ± 34.7 (92–111.9)	104.3 ± 38.1 (93.5–115.1)
	**M2**	5.4 ± 1.9 (4.8–5.9)	5.7 ± 1.9 (5.2–6.3)	5.2 ± 1.9 (4.7–5.8)	98.4 ± 43.4 (86.1–110.8)	105.4 ± 33.2 (95.7–115)	97.5 ± 32 (88.4–106.6)
	**M3**	5.6 ± 2.3 (4.9–6.2)	5.7 ± 1.7 (5.2–6.2)	5.4 ± 2.2 (4.8–6)	97.2 ± 31.4 (88.2–106.1)	108.5 ± 39.3 (97–120.1)	102.7 ± 40.8 (91.1–114.4)
**Cheek**	**T0**	3.9 ± 1.3 (3.5–4.3)	4 ± 1.2 (3.7–4.4)	3.9 ± 1 (3.6–4.1)	100 ± 0 (100–100)	100 ± 0 (100–100)	100 ± 0 (100–100)
	**M1**	3.8 ± 1.3 (3.4–4.2)	4 ± 1.3 (3.6–4.3)	3.9 ± 1.5 (3.4–4.3)	99.2 ± 21.5 (93.1–105.3)	106.5 ± 50.1 (92.1–120.9)	98.8 ± 21.2 (92.7–104.8)
	**M2**	3.8 ± 1.5 (3.4–4.2)	4 ± 1.4 (3.6–4.4)	3.8 ± 1.5 (3.4–4.3)	98.5 ± 31.2 (89.7–107.4)	106.4 ± 49.6 (92–120.8)	100.5 ± 30.2 (91.9–109)
	**M3**	3.9 ± 1.3 (3.6–4.3)	4.1 ± 1.6 (3.6–4.6)	3.9 ± 1.3 (3.6–4.3)	103.3 ± 24.8 (96.3–110.4)	107.8 ± 47.7 (93.8–121.8)	103.9 ± 27.1 (96.1–111.7)
**NMF – TOTAL**							
**Site**	**Time point**	**ng/mL**			**%T0 mean ± SD (95% CI)**		
		**Placebo**	**SH60**	**SH120**	**Placebo**	**SH60**	**SH120**
**Forearm**	**T0**	9754.5 ± 2430.5 (9032.8–10476.3)	10966.7 ± 3055.8 (10089–11844.4)	9977.2 ± 2456.6 (9271.6–10682.8)	100 ± 0 (100–100)	100 ± 0 (100–100)	100 ± 0 (100–100)
	**M1**	9767.6 ± 2127.7 (9120.7–10414.4)	11265.8 ± 3221.1 (10330–12201.1)	10706.8 ± 2595.3 (9953.2–11460.4)	107.4 ± 42.6 (94.4–120.3)	106.2 ± 26.9 (98.3–114)	112.4 ± 33 (102.9–122)
	**M2**	9595.9 ± 2185.4 (8939.3–10252.5)	10567.2 ± 2694.6 (9776.1–11358.4)	10302.2 ± 2429.4 (9588.9–11015.5)	103.1 ± 29.9 (94.2–112.1)	101.8 ± 35.7 (91.4–112.3)	107.2 ± 38.7 (95.8–118.6)

	M3	9432.2 ± 2256.7 (8746.1–10118.3)	10788.2 ± 3257.8 (9842.2–11734.1)	9998.5 ± 2636.7 (9232.9–10764.1)	97.6 ± 24.7 (90.1–105.2)	102.3 ± 32.8 (92.8–111.9)	103.6 ± 29.7 (95–112.3)
**NMF – FILAGGRIN-DERIVED**							
**Site**	**Time point**	**ng/mL**			**%T0 mean ± SD (95% CI)**		
		**Placebo**	**SH60**	**SH120**	**Placebo**	**SH60**	**SH120**
**Forearm**	**T0**	7293.5 ± 1953.9 (6713.3–7873.8)	7885.1 ± 2379.2 (7201.7–8568.5)	7373.2 ± 1926.6 (6819.8–7926.5)	100 ± 0 (100–100)	100 ± 0 (100–100)	100 ± 0 (100–100)
	**M1**	7544 ± 1755.7 (7010.2–8077.8)	8408.9 ± 2454.7 (7696.2–9121.7)	8121.1 ± 2011.3 (7537.1–8705.1)	110.9 ± 41.1 (98.4–123.4)	111.8 ± 28.6 (103.5–120.1)	117.8 ± 43.5 (105.2–130.4)
	**M2**	7510.9 ± 1859.4 (6952.3–8069.5)	7778.5 ± 1846.9 (7236.2–8320.8)	7985.8 ± 1956.6 (7411.3–8560.2)	110.4 ± 33.8 (100.1–120.6)	104 ± 29.7 (95.3–112.7)	115.4 ± 52.7 (99.9–130.9)
	**M3**	7432.2 ± 1667.2 (6925.4–7939.1)	8179.1 ± 2269.6 (7520.1–8838.2)	7749.4 ± 2028.7 (7160.3–8338.5)	103.7 ± 26.6 (95.7–111.8)	109.4 ± 35.5 (99.1–119.7)	111.1 ± 42.1 (98.8–123.3)
**NMF – UREA**							
**Site**	**Time point**	**ng/mL**			**%T0 mean ± SD (95% CI)**		
		**Placebo**	**SH60**	**SH120**	**Placebo**	**SH60**	**SH120**
**Forearm**	**T0**	1432.3 ± 962.8 (1146.3–1718.2)	1975 ± 1409.9 (1570.1–2380)	1553.6 ± 890.2 (1297.9–1809.3)	100 ± 0 (100–100)	100 ± 0 (100–100)	100 ± 0 (100–100)
	**M1**	1131.8 ± 715.3 (916.9–1346.7)	1649.9 ± 1226.2 (1293.9–2006)	1452.4 ± 947.3 (1177.4–1727.5)	108.8 ± 115.3 (74.1–143.4)	98.8 ± 77.1 (76.4–121.1)	110.2 ± 78.7 (87.4–133.1)
	**M2**	1007.2 ± 626.7 (818.9–1195.5)	1631.5 ± 1564.1 (1172.3–2090.8)	1187.8 ± 636.2 (1001–1374.6)	86.2 ± 64.5 (66.8–105.5)	102.5 ± 106.9 (71.1–133.9)	84.9 ± 45.7 (71.5–98.3)
	**M3**	973 ± 679.7 (766.3–1179.6)	1445.2 ± 1382.6 (1043.7–1846.6)	1125 ± 677.7 (928.2–1321.7)	84.9 ± 69 (64–105.9)	82 ± 59.5 (64.7–99.3)	82.2 ± 38.1 (71.2–93.3)
**NMF – SERINE**							
**Site**	**Time point**	**ng/mL**			**%T0 mean ± SD (95% CI)**		
		**Placebo**	**SH60**	**SH120**	**Placebo**	**SH60**	**SH120**
**Forearm**	**T0**	1855.8 ± 653.9 (1661.6–2050)	2037.5 ± 815 (1803.4–2271.6)	1884.8 ± 652.5 (1697.4–2072.2)	100 ± 0 (100–100)	100 ± 0 (100–100)	100 ± 0 (100–100)
	**M1**	1917.9 ± 562.6 (1748.8–2086.9)	2216.2 ± 850.6 (1969.3–2463.2)	2126.3 ± 718.3 (1917.7–2334.9)	119.7 ± 69 (99–140.4)	118.6 ± 40.3 (106.9–130.3)	127.1 ± 71 (106.5–147.8)
	**M2**	1914.7 ± 625.9 (1726.7-2102.7)	1995 ± 646.3 (1805.2–2184.8)	2069.7 ± 668.3 (1873.5–2266)	115.2 ± 54 (99–131.4)	107.9 ± 40.9 (95.9–119.9)	126.8 ± 107.5 (95.2–158.4)
	**M3**	1905.7 ± 556.5 (1736.5–2074.9)	2128.1 ± 763.8 (1906.4–2349.9)	2004.9 ± 684.2 (1806.2–2203.6)	108.5 ± 40 (96.3–120.6)	115.8 ± 50.3 (101.2–130.4)	118.6 ± 71.2 (97.9–139.2)
**NMF – GLYCINE**							
**Site**	**Time point**	**ng/mL**			**%T0 mean ± SD (95% CI)**		
		**Placebo**	**SH60**	**SH120**	**Placebo**	**SH60**	**SH120**
**Forearm**	**T0**	802.2 ± 245.9 (729.1–875.2)	874.3 ± 314.9 (783.8–964.7)	808.7 ± 260.5 (733.9–883.5)	100 ± 0 (100–100)	100 ± 0 (100–100)	100 ± 0 (100–100)
	**M1**	836.5 ± 233.5 (766.3–906.6)	920.1 ± 308.1 (830.7–1009.6)	905.9 ± 271.9 (826.9–984.8)	114.3 ± 49.9 (99.3–129.3)	112.7 ± 33.6 (103–122.5)	122.5 ± 50.4 (107.8–137.1)
	**M2**	816.6 ± 235.6 (745.8–887.4)	837.4 ± 256.6 (762.1–912.7)	886.8 ± 258.2 (811–962.6)	110.2 ± 40.8 (98–122.5)	102.4 ± 33 (92.8–112.1)	119.6 ± 63.4 (100.9–138.2)
	**M3**	809.1 ± 196.8 (749.3–869)	882.8 ± 275.4 (802.8–962.8)	868.1 ± 258.6 (793.1–943.2)	105.5 ± 36.3 (94.5–116.6)	109 ± 39.6 (97.4–120.5)	116.4 ± 52.4 (101.1–131.6)
**NMF – PCA**							
**Site**	**Time point**	**ng/mL**			**%T0 mean ± SD (95% CI)**		
		**Placebo**	**SH60**	**SH120**	**Placebo**	**SH60**	**SH120**
**Forearm**	**T0**	257 ± 30.8 (247.8–266.1)	264.8 ± 48.3 (250.9–278.6)	253.4 ± 33.2 (243.8–262.9)	100 ± 0 (100–100)	100 ± 0 (100–100)	100 ± 0 (100–100)
	**M1**	267.3 ± 33.4 (257.3–277.4)	281.2 ± 46.3 (267.7–294.6)	270.1 ± 36.4 (259.5–280.6)	105.8 ± 18 (100.4–111.2)	107.6 ± 15.2 (103.2–112)	108.2 ± 16.8 (103.3–113)
	**M2**	265.3 ± 34.4 (255–275.6)	265.5 ± 37.9 (254.4–276.6)	268.9 ± 32.9 (259.3–278.6)	104.3 ± 15.2 (99.7–108.8)	102.2 ± 17.1 (97.1–107.2)	107 ± 16.7 (102.1–111.9)
	**M3**	264.3 ± 32.2 (254.5–274.1)	275.5 ± 41.1 (263.5–287.4)	268.4 ± 38.2 (257.3–279.5)	100.4 ± 20.4 (94.3–106.5)	105.8 ± 17.6 (100.7–110.9)	106.9 ± 16.3 (102.2–111.6)

Table S6. *P* values for between-group comparisons (SH60 and SH120 vs. placebo) at each time point for all skin parameters. Statistically significant values ($p < 0.05$) obtained by unpaired t-test are shown in **bold**. Statistically significant values obtained by Mann–Whitney U test are shown in **bold** with an asterisk (*).

**HYDRATION**			
**Site**	**Time point**	***p* value**	
		**SH60**	**SH120**
**Forehead**	**M1**	0.376	0.234
	**M2**	0.250	0.425
	**M3**	**0.009**	**0.047**
**Cheek**	**M1**	0.250	0.427
	**M2**	0.363	0.155
	**M3**	**0.018**	**0.010**
**Forearm**	**M1**	**0.023**	0.390
	**M2**	0.330	0.234
	**M3**	0.052	0.312
**TEWL**			
**Site**	**Time point**	***p* value**	
		**SH60**	**SH120**
**Forehead**	**M1**	0.347	0.211
	**M2**	0.246	0.363
	**M3**	**0.018**	**0.002**
**Cheek**	**M1**	0.189	0.310
	**M2**	0.159	0.186
	**M3**	**0.016**	**0.003**
**SEBUM**			
**Site**	**Time point**	***p* value**	
		**SH60**	**SH120**
**Forehead**	**M1**	0.210	0.408
	**M2**	0.302	0.165
	**M3**	0.315	0.120
**Cheek**	**M1**	0.251	0.235
	**M2**	0.490	0.110
	**M3**	0.086	**0.020***
**WRINKLE DEPTH**			
**Site**	**Time point**	***p* value**	
		**SH60**	**SH120**
**Crow's feet**	**M1**	**0.002**	**0.001**
	**M2**	**0.004**	**0.000**
	**M3**	**0.005**	**0.000**
**DERMAL DENSITY (COLLAGEN LEVEL)**			
**Site**	**Time point**	***p* value**	
		**SH60**	**SH120**
**Forehead**	**M1**	0.394	0.149
	**M2**	0.285	**0.0002***
	**M3**	0.335	**0.024***
**EPIDERMAL THICKNESS**			
**Site**	**Time point**	***p* value**	
		**SH60**	**SH120**
**Forehead**	**M1**	0.207	0.090
	**M2**	0.441	0.324
	**M3**	0.449	**0.020**
**ELASTICITY – R0**			
**Site**	**Time point**	***p* value**	
		**SH60**	**SH120**
**Forehead**	**M1**	0.180	0.310
	**M2**	**0.034**	0.113
	**M3**	0.130	**0.049***
**Cheek**	**M1**	**0.021**	0.345
	**M2**	0.383	0.361
	**M3**	0.118	0.261
**ELASTICITY – R1**			
**Site**	**Time point**	***p* value**	
		**SH60**	**SH120**
**Forehead**	**M1**	0.222	0.472
	**M2**	0.060	0.205
	**M3**	0.198	0.110
**Cheek**	**M1**	0.094	0.305
	**M2**	0.207	0.327
	**M3**	0.292	0.308
**ELASTICITY – R2**			
**Site**	**Time point**	***p* value**	
		**SH60**	**SH120**
**Forehead**	**M1**	0.140	0.084
	**M2**	0.319	0.319
	**M3**	0.370	0.500
**Cheek**	**M1**	0.447	0.444
	**M2**	0.093	0.236
	**M3**	**0.041**	0.472
**ELASTICITY – R3**			
**Site**	**Time point**	***p* value**	

		**SH60**	**SH120**
**Forehead**	**M1**	0.200	0.429
	**M2**	**0.027**	0.139
	**M3**	0.160	**0.035***
**Cheek**	**M1**	**0.034**	0.389
	**M2**	0.408	0.444
	**M3**	0.146	0.302
**ELASTICITY – R4**			
**Site**	**Time point**	***p* value**	
		**SH60**	**SH120**
**Forehead**	**M1**	0.375	0.417
	**M2**	**0.041**	0.205
	**M3**	0.287	**0.058***
**Cheek**	**M1**	0.053	0.434
	**M2**	0.371	0.394
	**M3**	0.281	0.360
**ELASTICITY – R5**			
**Site**	**Time point**	***p* value**	
		**SH60**	**SH120**
**Forehead**	**M1**	0.360	0.087
	**M2**	0.270	0.351
	**M3**	0.115	**0.016**
**Cheek**	**M1**	0.494	0.302
	**M2**	0.385	0.370
	**M3**	0.247	0.407
**ELASTICITY – R6**			
**Site**	**Time point**	***p* value**	
		**SH60**	**SH120**
**Forehead**	**M1**	0.408	0.246
	**M2**	0.281	0.324
	**M3**	**0.043**	**0.005**
**Cheek**	**M1**	0.386	0.170
	**M2**	0.169	0.148
	**M3**	0.339	0.052
**ELASTICITY – R7**			
**Site**	**Time point**	***p* value**	
		**SH60**	**SH120**
**Forehead**	**M1**	0.281	0.080
	**M2**	0.320	0.365
	**M3**	0.163	**0.017**
**Cheek**	**M1**	0.492	0.463
	**M2**	0.483	0.424
	**M3**	0.147	0.326
**ELASTICITY – R9**			
**Site**	**Time point**	***p* value**	
		**SH60**	**SH120**
**Forehead**	**M1**	0.399	0.371
	**M2**	0.143	0.339
	**M3**	0.308	0.179
**Cheek**	**M1**	0.451	0.275
	**M2**	0.260	0.181
	**M3**	0.194	0.158
**REDNESS AREAS**			
**Site**	**Time point**	***p* value**	
		**SH60**	**SH120**
**Forehead**	**M1**	0.054	0.162
	**M2**	0.401	0.352
	**M3**	0.280	0.178
**Cheek**	**M1**	0.498	0.197
	**M2**	0.309	0.342
	**M3**	0.308	0.420
**PORE SIZE**			
**Site**	**Time point**	***p* value**	
		**SH60**	**SH120**
**Forehead**	**M1**	0.139	0.322
	**M2**	0.416	0.336
	**M3**	0.278	0.118
**Cheek**	**M1**	0.386	**0.005**
	**M2**	0.450	0.172
	**M3**	0.195	0.334
**ERYTHEMA INDEX**			
**Site**	**Time point**	***p* value**	
		**SH60**	**SH120**
**Forehead**	**M1**	0.205	0.303
	**M2**	0.305	0.391
	**M3**	0.423	0.330
**Cheek**	**M1**	0.273	0.315
	**M2**	0.174	0.355
	**M3**	0.255	0.383
**MELANIN INDEX**			
**Site**	**Time point**	***p* value**	

		SH60	SH120
**Forehead**	**M1**	0.065	0.147
	**M2**	0.239	0.301
	**M3**	0.245	0.327
**Cheek**	**M1**	0.139	0.337
	**M2**	0.128	0.258
	**M3**	0.147	0.449
**ITA°**			
**Site**	**Time point**	***p* value**	
		**SH60**	**SH120**
**Forehead**	**M1**	0.305	0.305
	**M2**	0.223	0.447
	**M3**	0.258	0.442
**Cheek**	**M1**	0.255	0.077
	**M2**	0.486	0.197
	**M3**	0.399	0.363
**a* VALUE**			
**Site**	**Time point**	***p* value**	
		**SH60**	**SH120**
**Forehead**	**M1**	0.428	0.411
	**M2**	0.420	0.315
	**M3**	0.228	0.358
**Cheek**	**M1**	0.492	0.325
	**M2**	0.416	0.409
	**M3**	0.389	0.381
**b* VALUE**			
**Site**	**Time point**	***p* value**	
		**SH60**	**SH120**
**Forehead**	**M1**	0.250	0.187
	**M2**	0.324	0.277
	**M3**	0.343	0.081
**Cheek**	**M1**	0.106	0.138
	**M2**	0.205	0.380
	**M3**	0.312	0.399
**GLOSS**			
**Site**	**Time point**	***p* value**	
		**SH60**	**SH120**
**Forehead**	**M1**	0.347	0.244
	**M2**	0.188	0.451
	**M3**	0.059	0.223
**Cheek**	**M1**	0.173	0.460
	**M2**	0.175	0.377
	**M3**	0.279	0.457
**NMF – TOTAL**			
**Site**	**Time point**	***p* value**	
		**SH60**	**SH120**
**Forearm**	**M1**	0.435	0.262
	**M2**	0.425	0.287
	**M3**	0.222	0.150
**NMF – FILAGGRIN-DERIVED**			
**Site**	**Time point**	***p* value**	
		**SH60**	**SH120**
**Forearm**	**M1**	0.452	0.217
	**M2**	0.171	0.295
	**M3**	0.195	0.163
**NMF – UREA**			
**Site**	**Time point**	***p* value**	
		**SH60**	**SH120**
**Forearm**	**M1**	0.313	0.472
	**M2**	0.190	0.457
	**M3**	0.414	0.410
**NMF – SERINE**			
**Site**	**Time point**	***p* value**	
		**SH60**	**SH120**
**Forearm**	**M1**	0.462	0.305
	**M2**	0.232	0.259
	**M3**	0.220	0.200
**NMF – GLYCINE**			
**Site**	**Time point**	***p* value**	
		**SH60**	**SH120**
**Forearm**	**M1**	0.428	0.218
	**M2**	0.158	0.203
	**M3**	0.334	0.128
**NMF – PCA**			
**Site**	**Time point**	***p* value**	
		**SH60**	**SH120**
**Forearm**	**M1**	0.301	0.254
	**M2**	0.266	0.206
	**M3**	0.087	**0.046**

Table S7. Complete aggregated dataset of participant-reported current state of the skin parameters, assessed via questionnaire using a 5-point Likert-type scale. Values are presented as mean ± SD (95% CI). Time points: T0 = baseline, M0.5 = 2 weeks, M1 = 1 month, etc.

Current state of:	Time point	Mean ± SD (95% CI)		
		Placebo	SH60	SH120
Hydration (1 – hydrated, 5 – dry)	T0	3.2 ± 0.8 (2.9–3.5)	3.0 ± 1.0 (2.7–3.4)	3.1 ± 0.9 (2.8–3.4)
	M0.5	2.8 ± 0.8 (2.6–3.0)	2.8 ± 0.8 (2.6–3.0)	3.0 ± 0.7 (2.8–3.2)
	M1	2.8 ± 0.8 (2.6–3.1)	2.8 ± 0.8 (2.5–3.0)	2.8 ± 0.8 (2.6–3.0)
	M1.5	2.8 ± 0.6 (2.6–3.0)	2.7 ± 0.7 (2.5–2.9)	2.9 ± 0.7 (2.7–3.1)
	M2	2.6 ± 0.7 (2.4–2.8)	2.5 ± 0.6 (2.3–2.7)	2.6 ± 0.7 (2.4–2.9)
	M2.5	2.6 ± 0.8 (2.4–2.8)	2.4 ± 0.7 (2.1–2.6)	2.6 ± 0.7 (2.3–2.8)
	M3	2.5 ± 0.7 (2.3–2.7)	2.6 ± 0.7 (2.4–2.8)	2.4 ± 0.6 (2.3–2.6)
Sebum on the forehead (1 – dry, 5 – oily)	T0	3.4 ± 0.8 (3.1–3.7)	3.3 ± 0.9 (3.0–3.7)	3.5 ± 1.1 (3.1–3.9)
	M0.5	3.1 ± 0.8 (2.9–3.4)	3.0 ± 0.7 (2.8–3.2)	3.2 ± 0.8 (2.9–3.4)
	M1	2.9 ± 0.8 (2.7–3.1)	2.7 ± 0.7 (2.5–2.9)	2.8 ± 0.9 (2.6–3.0)
	M1.5	2.9 ± 0.7 (2.7–3.1)	2.8 ± 0.6 (2.6–3.0)	2.9 ± 0.7 (2.7–3.2)
	M2	2.9 ± 0.7 (2.7–3.1)	2.9 ± 0.6 (2.7–3.1)	2.8 ± 0.9 (2.5–3.0)
	M2.5	2.8 ± 0.7 (2.6–3.0)	2.7 ± 0.5 (2.6–2.9)	3.1 ± 0.7 (2.8–3.3)
	M3	2.8 ± 0.7 (2.6–2.9)	2.5 ± 0.7 (2.2–2.7)	2.7 ± 0.7 (2.4–2.9)
Sebum on the cheek (1 – dry, 5 – oily)	T0	2.7 ± 0.8 (2.4–3.0)	2.5 ± 0.9 (2.2–2.8)	2.7 ± 0.9 (2.4–3.1)
	M0.5	2.5 ± 0.7 (2.3–2.7)	2.5 ± 0.7 (2.3–2.7)	2.8 ± 0.8 (2.6–3.0)
	M1	2.5 ± 0.7 (2.3–2.7)	2.3 ± 0.8 (2.1–2.5)	2.6 ± 0.7 (2.3–2.8)
	M1.5	2.6 ± 0.6 (2.4–2.8)	2.5 ± 0.7 (2.3–2.7)	2.7 ± 0.7 (2.5–2.9)
	M2	2.6 ± 0.7 (2.4–2.8)	2.5 ± 0.7 (2.3–2.7)	2.6 ± 0.7 (2.4–2.8)
	M2.5	2.6 ± 0.6 (2.4–2.8)	2.5 ± 0.7 (2.3–2.7)	2.7 ± 0.8 (2.4–2.9)
	M3	2.5 ± 0.5 (2.4–2.7)	2.3 ± 0.7 (2.1–2.5)	2.4 ± 0.7 (2.2–2.6)
Roughness (1 – smooth, 5 – rough)	T0	3.5 ± 0.7 (3.2–3.7)	3.4 ± 0.7 (3.2–3.7)	3.3 ± 0.9 (2.9–3.6)
	M0.5	2.6 ± 0.7 (2.4–2.8)	2.5 ± 0.9 (2.2–2.7)	2.5 ± 0.7 (2.3–2.7)
	M1	2.6 ± 0.8 (2.4–2.8)	2.4 ± 0.8 (2.2–2.7)	2.6 ± 0.8 (2.3–2.8)
	M1.5	2.3 ± 0.7 (2.1–2.5)	2.3 ± 0.8 (2.1–2.5)	2.6 ± 0.7 (2.3–2.8)
	M2	2.4 ± 0.7 (2.2–2.6)	2.3 ± 0.7 (2.1–2.5)	2.4 ± 0.7 (2.1–2.6)
	M2.5	2.2 ± 0.7 (2.0–2.4)	2.2 ± 0.9 (1.9–2.5)	2.4 ± 0.7 (2.2–2.6)
	M3	2.3 ± 0.7 (2.1–2.5)	2.3 ± 0.7 (2.1–2.5)	2.4 ± 0.6 (2.2–2.5)
Elasticity (1 – good/high, 5 – bad/low)	T0	2.4 ± 1.0 (2.0–2.7)	2.5 ± 0.9 (2.2–2.9)	2.4 ± 1.0 (2.0–2.7)
	M0.5	2.6 ± 0.8 (2.4–2.8)	2.5 ± 0.9 (2.3–2.8)	2.6 ± 0.7 (2.4–2.8)
	M1	2.5 ± 0.7 (2.3–2.7)	2.4 ± 0.9 (2.2–2.7)	2.4 ± 0.7 (2.2–2.6)
	M1.5	2.4 ± 0.7 (2.2–2.6)	2.3 ± 0.8 (2.1–2.5)	2.6 ± 0.7 (2.3–2.8)
	M2	2.4 ± 0.6 (2.2–2.6)	2.4 ± 0.8 (2.1–2.6)	2.5 ± 0.8 (2.3–2.7)
	M2.5	2.4 ± 0.8 (2.2–2.6)	2.2 ± 0.8 (1.9–2.4)	2.4 ± 0.7 (2.2–2.6)
	M3	2.3 ± 0.7 (2.1–2.5)	2.3 ± 0.9 (2.0–2.5)	2.2 ± 0.7 (2.0–2.4)
Number of wrinkles (1 – none, 5 – many)	T0	2.5 ± 1.0 (2.1–2.8)	2.4 ± 0.9 (2.1–2.7)	2.6 ± 1.0 (2.2–2.9)
	M0.5	2.6 ± 0.9 (2.3–2.8)	2.4 ± 0.9 (2.1–2.6)	2.6 ± 0.7 (2.5–2.8)
	M1	2.6 ± 1.0 (2.3–2.8)	2.4 ± 0.9 (2.2–2.7)	2.5 ± 0.9 (2.3–2.8)
	M1.5	2.5 ± 0.8 (2.2–2.7)	2.3 ± 0.7 (2.1–2.5)	2.6 ± 0.7 (2.4–2.8)
	M2	2.6 ± 1.0 (2.3–2.8)	2.4 ± 0.9 (2.1–2.6)	2.4 ± 0.9 (2.2–2.6)
	M2.5	2.4 ± 0.8 (2.2–2.6)	2.3 ± 0.8 (2.0–2.5)	2.4 ± 0.7 (2.2–2.6)
	M3	2.5 ± 0.8 (2.2–2.7)	2.1 ± 0.8 (1.9–2.4)	2.3 ± 0.8 (2.1–2.5)
Depth of wrinkles (1 –none, 5 – very deep)	T0	2.3 ± 0.9 (1.9–2.6)	2.2 ± 0.8 (1.9–2.5)	2.4 ± 1.0 (2.0–2.8)
	M0.5	2.4 ± 0.9 (2.1–2.6)	2.3 ± 0.9 (2.1–2.6)	2.5 ± 0.7 (2.3–2.7)
	M1	2.4 ± 0.8 (2.1–2.6)	2.2 ± 0.8 (2.0–2.4)	2.3 ± 0.8 (2.1–2.5)
	M1.5	2.3 ± 0.7 (2.1–2.5)	2.2 ± 0.8 (2.0–2.4)	2.6 ± 0.6 (2.4–2.8)
	M2	2.4 ± 0.8 (2.1–2.6)	2.2 ± 0.8 (1.9–2.4)	2.3 ± 0.8 (2.0–2.5)
	M2.5	2.3 ± 0.8 (2.1–2.5)	2.2 ± 0.8 (1.9–2.4)	2.3 ± 0.7 (2.1–2.6)
	M3	2.4 ± 0.7 (2.2–2.6)	2.0 ± 0.7 (1.8–2.2)	2.2 ± 0.7 (2.0–2.4)
Skin sensitivity (1 – low, 5 – high)	T0	1.7 ± 1.0 (1.4–2.1)	2.1 ± 1.1 (1.7–2.5)	2.3 ± 1.2 (1.8–2.7)
	M0.5	2.0 ± 1.0 (1.7–2.3)	2.1 ± 0.9 (1.8–2.4)	2.3 ± 1.1 (2.0–2.6)
	M1	1.8 ± 0.9 (1.5–2.0)	1.9 ± 1.1 (1.6–2.2)	2.2 ± 1.2 (1.8–2.5)
	M1.5	2.1 ± 0.9 (1.8–2.4)	2.2 ± 0.8 (1.9–2.4)	2.2 ± 0.9 (2.0–2.5)
	M2	1.9 ± 1.0 (1.6–2.2)	1.7 ± 0.7 (1.5–1.9)	1.9 ± 1.0 (1.7–2.2)
	M2.5	2.0 ± 0.9 (1.7–2.3)	2.1 ± 0.9 (1.8–2.3)	2.3 ± 1.0 (2.0–2.6)
	M3	1.9 ± 1.0 (1.6–2.2)	1.9 ± 0.9 (1.6–2.2)	2.0 ± 1.1 (1.7–2.3)

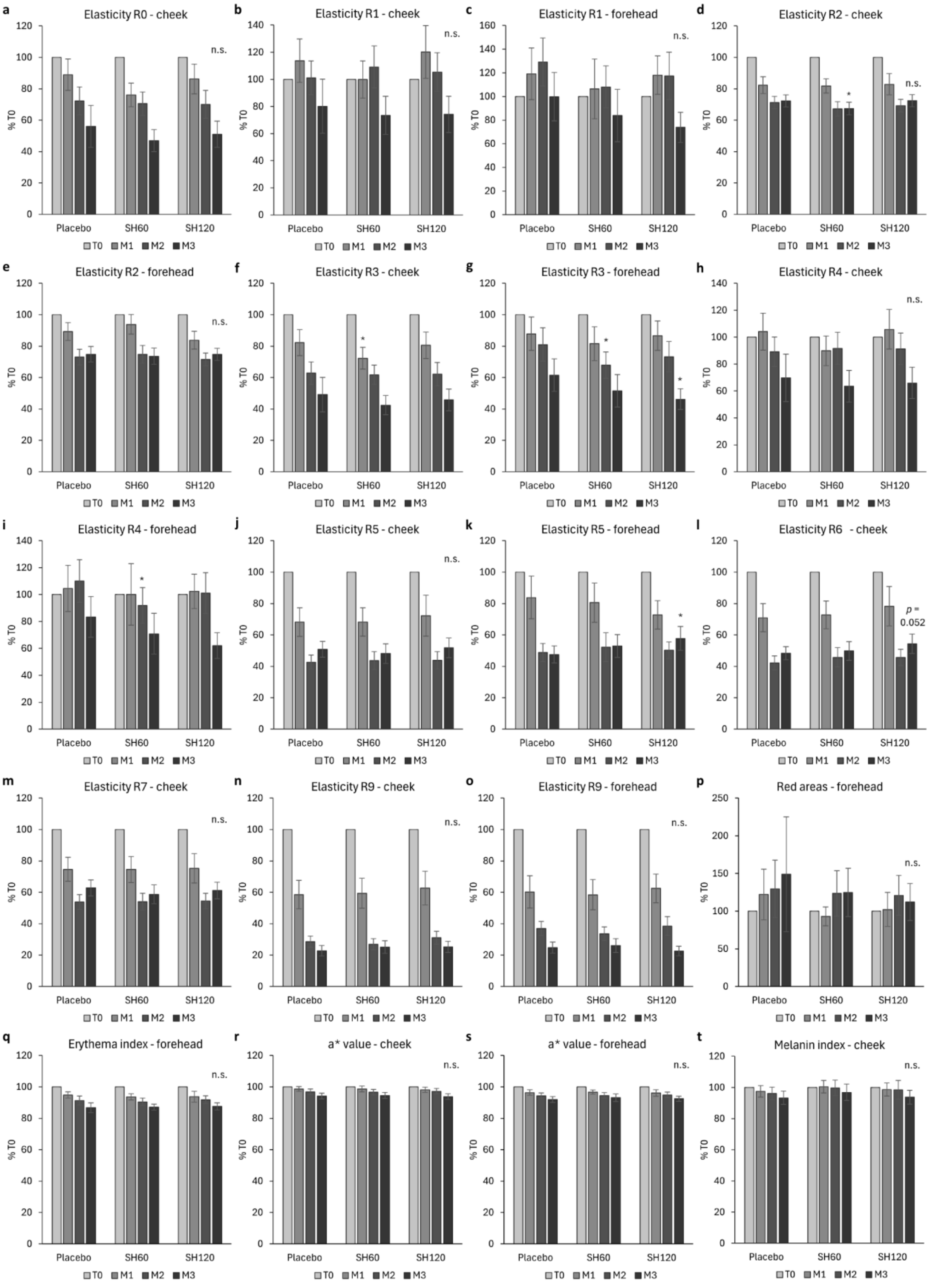
a
Elasticity R0 - cheek
n.s.
b
Elasticity R1 - cheek
n.s.
c
Elasticity R1 - forehead
n.s.
d
Elasticity R2 - cheek
*
n.s.
e
Elasticity R2 - forehead
n.s.
f
Elasticity R3 - cheek
*
g
Elasticity R3 - forehead
*
*
h
Elasticity R4 - cheek
n.s.
i
Elasticity R4 - forehead
*
j
Elasticity R5 - cheek
n.s.
k
Elasticity R5 - forehead
*
l
Elasticity R6 - cheek
p = 0.052
m
Elasticity R7 - cheek
n.s.
n
Elasticity R9 - cheek
n.s.
o
Elasticity R9 - forehead
n.s.
p
Red areas - forehead
n.s.
q
Erythema index - forehead
n.s.
r
a* value - cheek
n.s.
s
a* value - forehead
n.s.
t
Melanin index - cheek
n.s.
% T0
Placebo
SH60
SH120
T0
M1
M2
M3

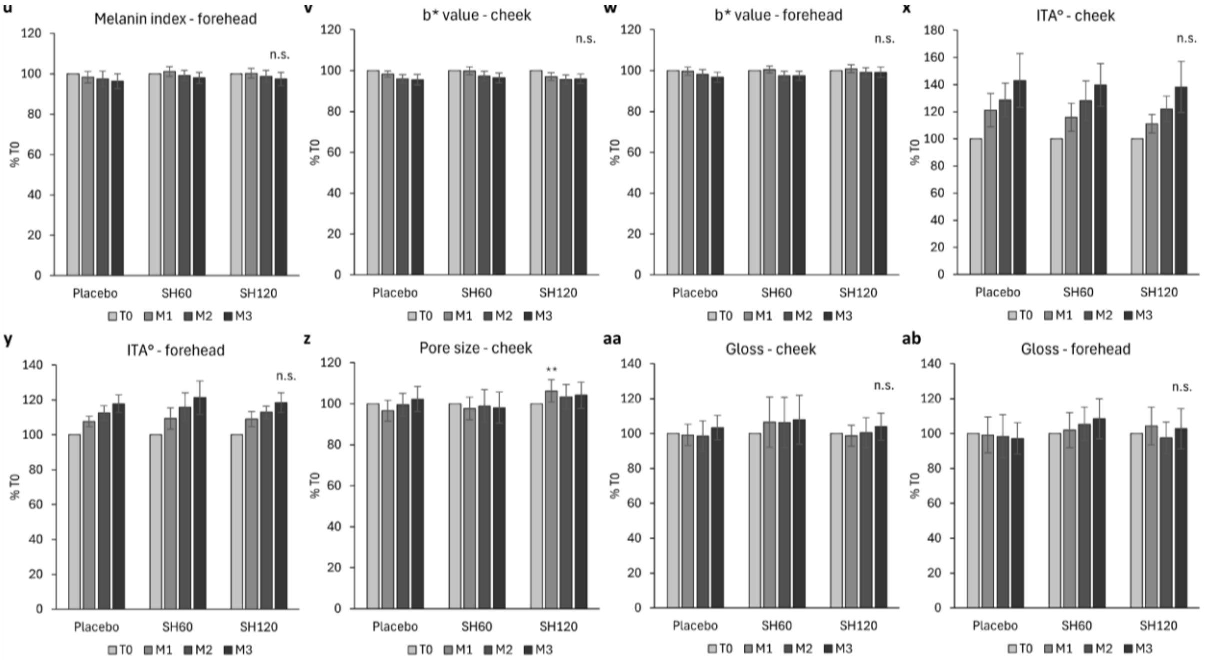

Figure S3. Effect of oral supplementation with SH60 and SH120 on selected skin parameters: (**a**) R0 on the cheek; (**b**) R1 on the cheek; (**c**) R1 on the forehead; (**d**) R2 on the cheek; (**e**) R2 on the forehead; (**f**) R3 on the cheek; (**g**) R3 on the forehead; (**h**) R4 on the cheek; (**i**) R4 on the forehead; (**j**) R5 on the cheek; (**k**) R5 on the forehead; (**l**) R6 on the cheek; (**m**) R7 on the cheek; (**n**) R9 on the cheek; (**o**) R9 on the forehead; (**p**) redness areas on the forehead; (**q**) erythema index on the forehead; (**r**) a* value on the cheek; (**s**) a* value on the forehead; (**t**) melanin index on the cheek; (**u**) melanin index on the forehead; (**v**) b* value on the cheek; (**w**) b* value on the forehead; (**x**) ITA° on the cheek; (**y**) ITA° on the forehead; (**z**) pore size on the cheek; (**aa**) gloss on the cheek; (**ab**) gloss on the forehead. Data are presented as percentage of baseline (%T0; mean ± 95% CI). *$p < 0.05$, **$p < 0.01$ vs. placebo.

Subjective assessment of the skin parameters (questionnaires)

The second set of questionnaire items assessed participants' perceived changes in skin parameters compared to baseline. **Figure S4** presents the proportion of participants who reported selected changes for each parameter throughout the study.

A progressively increasing number of participants perceived improved skin **hydration** in all study groups, including placebo, with approximately 40-50% reporting better hydration by the end of the intervention (**Figure S4A**). No differences between the intervention groups were noted. This trend suggests a strong expectation- or placebo-related effect.

In the case of skin **oiliness**, the majority of participants (>70%) indicated no change from baseline (**Figure S4B**). Only a small proportion (~15%) perceived a reduction in oiliness, with no differences between the intervention groups. Similar proportion (~10-15%) reported the opposite effect, i.e. increase in oiliness in placebo group. In this case, a slightly lower proportion of participants (~5-10%) reported this increase in the SH60 and SH120 groups.

Perceived improvement in skin **roughness** was also reported by a substantial proportion of participants across all groups (~30-40%, **Figure S4C**), again without evident differences between treatment and placebo groups, further supporting the presence of a placebo effect similar to that seen for hydration.

Perceived improvement in skin **elasticity** was reported by approximately 20-40% of participants at the end of the study (**Figure S4D**) with a slightly lower proportion in the SH120 group. Most participants noted no change, and only a negligible number (<2%) reported worsening of skin elasticity.

The proportion of participants reporting a reduction in **wrinkle number** and **depth** was around 20% and 30%, respectively, across all groups, with no marked differences between them (**Figure S4E, S4F**). The majority observed no change, and only a small minority (<4%) perceived an increase in either parameter.

Regarding **skin sensitivity**, the vast majority of participants (mostly >90%) noted no change throughout the study (**Figure S4G**). Only a small proportion in each group perceived their skin as less sensitive regardless of the intervention.

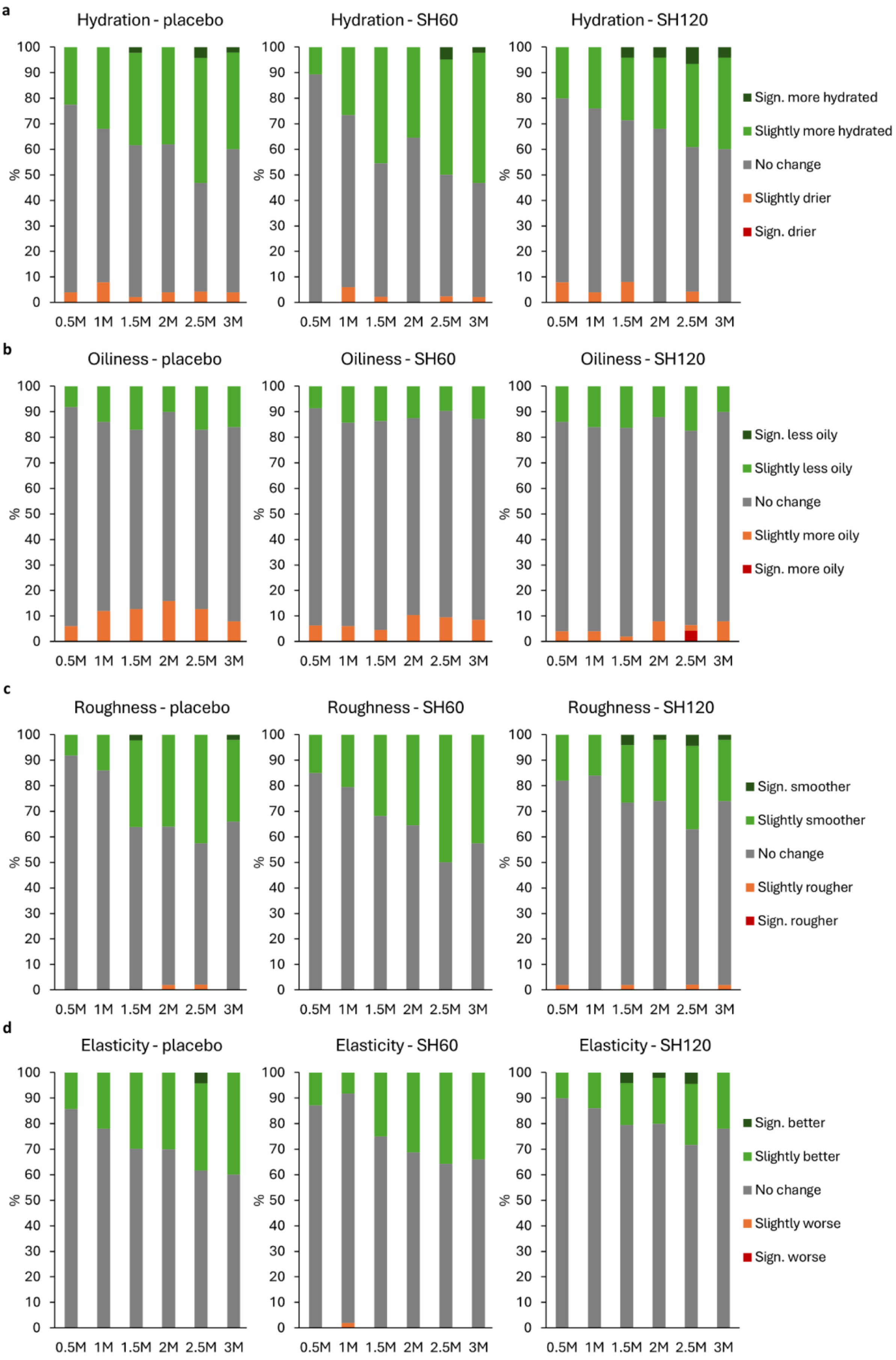
a
Hydration - placebo
Hydration - SH60
Hydration - SH120
Sign. more hydrated
Slightly more hydrated
No change
Slightly drier
Sign. drier
b
Oiliness - placebo
Oiliness - SH60
Oiliness - SH120
Sign. less oily
Slightly less oily
No change
Slightly more oily
Sign. more oily
c
Roughness - placebo
Roughness - SH60
Roughness - SH120
Sign. smoother
Slightly smoother
No change
Slightly rougher
Sign. rougher
d
Elasticity - placebo
Elasticity - SH60
Elasticity - SH120
Sign. better
Slightly better
No change
Slightly worse
Sign. worse
%
0 10 20 30 40 50 60 70 80 90 100
0.5M 1M 1.5M 2M 2.5M 3M

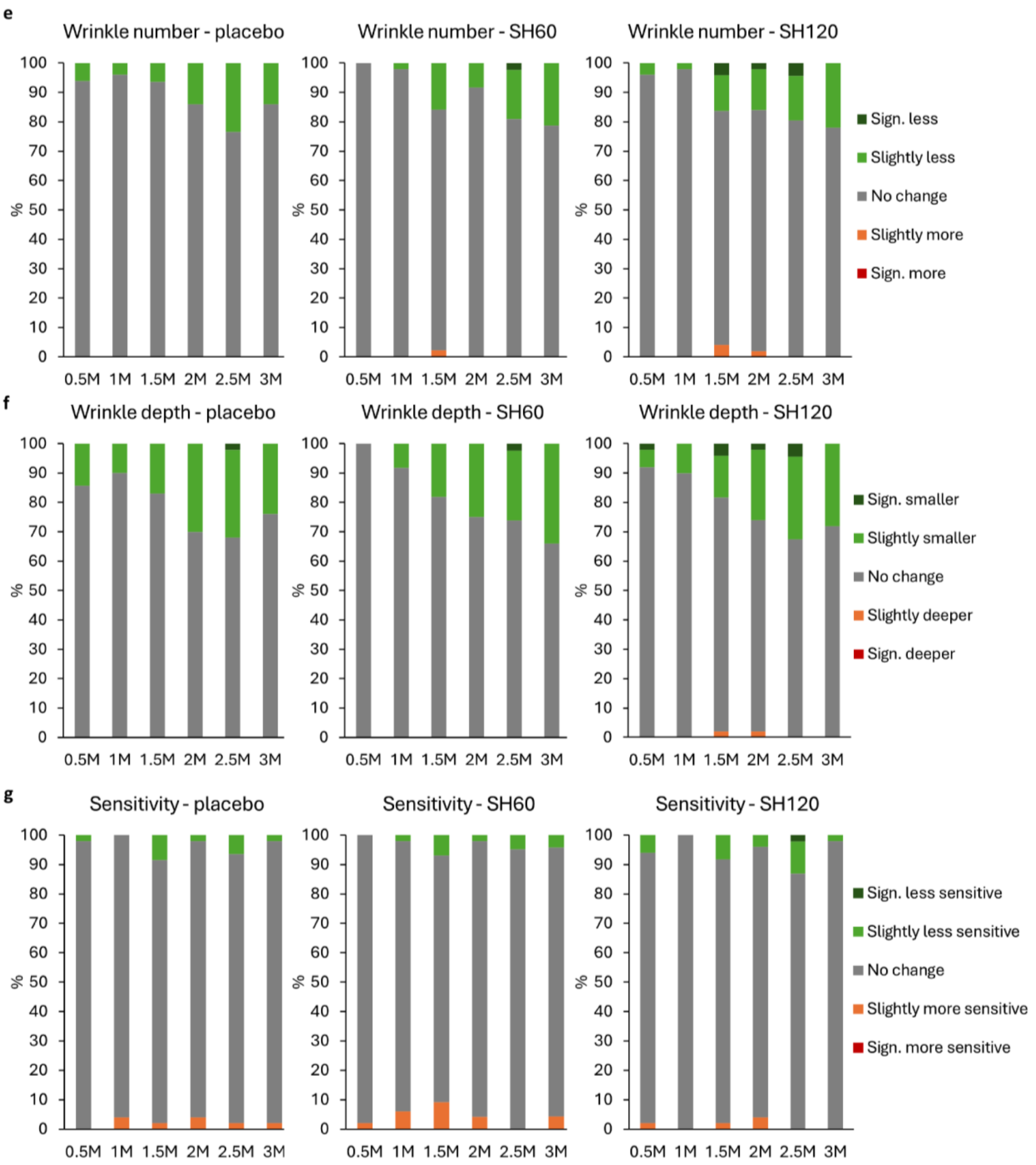

Figure S4. Self-reported perceived changes in skin parameters over time. Participants evaluated changes in their skin condition compared to baseline using a 5-point scale. The graphs show the percentage of participants reporting selected changes for each parameter and time point: (**a**) hydration; (**b**) oiliness; (**c**) roughness; (**d**) elasticity; (**e**) number of wrinkles; (**f**) depth of wrinkles; (**g**) sensitivity. Time points: T0 = baseline, M0.5 = 2 weeks, M1 = 1 month, etc.

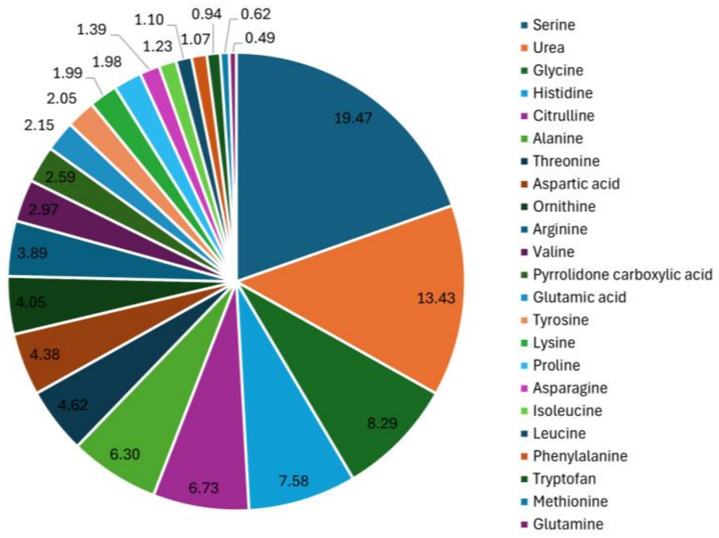

Figure S5. Relative composition of natural moisturizing factor (NMF) components in the stratum corneum of volar forearm skin analyzed in this study by LC-MS/MS. Pie chart shows the average proportion (%) of individual NMF-related molecules quantified across all collected samples.
